# Molecular genomic features associated with *in vitro* response of the NCI‐60 cancer cell line panel to natural products

**DOI:** 10.1002/1878-0261.12849

**Published:** 2020-11-24

**Authors:** Julia Krushkal, Simarjeet Negi, Laura M. Yee, Jason R. Evans, Tanja Grkovic, Alida Palmisano, Jianwen Fang, Hari Sankaran, Lisa M. McShane, Yingdong Zhao, Barry R. O'Keefe

**Affiliations:** ^1^ Biometric Research Program Division of Cancer Treatment and Diagnosis National Cancer Institute NIH Rockville MD USA; ^2^ Natural Products Branch Developmental Therapeutics Program Division of Cancer Treatment and Diagnosis National Cancer Institute Frederick MD USA; ^3^ Natural Products Support Group Frederick National Laboratory for Cancer Research Frederick MD USA; ^4^ General Dynamics Information Technology (GDIT) Falls Church VA USA; ^5^ Molecular Targets Program Center for Cancer Research National Cancer Institute Frederick MD USA

**Keywords:** cancer cell lines, DNA mutation, drug response, gene expression, natural products, single nucleotide variation

## Abstract

Natural products remain a significant source of anticancer chemotherapeutics. The search for targeted drugs for cancer treatment includes consideration of natural products, which may provide new opportunities for antitumor cytotoxicity as single agents or in combination therapy. We examined the association of molecular genomic features in the well‐characterized NCI‐60 cancer cell line panel with *in vitro* response to treatment with 1302 small molecules which included natural products, semisynthetic natural product derivatives, and synthetic compounds based on a natural product pharmacophore from the Developmental Therapeutics Program of the US National Cancer Institute's database. These compounds were obtained from a variety of plant, marine, and microbial species. Molecular information utilized for the analysis included expression measures for 23059 annotated transcripts, lncRNAs, and miRNAs, and data on protein‐changing single nucleotide variants in 211 cancer‐related genes. We found associations of expression of multiple genes including *SLFN11*, *CYP2J2*, *EPHX1*, *GPC1*, *ELF3*, and *MGMT* involved in *DNA* damage repair, *NOTCH* family members, *ABC* and *SLC* transporters, and both mutations in tyrosine kinases and *BRAF* V600E with NCI‐60 responses to specific categories of natural products. Hierarchical clustering identified groups of natural products, which correlated with a specific mechanism of action. Specifically, several natural product clusters were associated with *SLFN11* gene expression, suggesting that potential action of these compounds may involve DNA damage. The associations between gene expression or genome alterations of functionally relevant genes with the response of cancer cells to natural products provide new information about potential mechanisms of action of these identified clusters of compounds with potentially similar biological effects. This information will assist in future drug discovery and in design of new targeted cancer chemotherapy agents.

## Graphical abstract

Natural products provide new opportunities for anticancer chemotherapeutics. We examined the associations of molecular features in the NCI‐60 cancer cell lines with response to 1302 plant, marine, and microbial compounds. Expression or mutations in multiple genes were associated with treatment responses, suggesting potential mechanisms of action of natural compounds. This information will assist in future design of new chemotherapy agents.

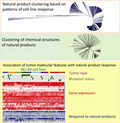

**Abbreviations**
ABC: ATP‐binding cassette
CNS: central nervous system
DTP: Developmental Therapeutics Program
MDR: multidrug resistance
NCI: National Cancer Institute
PI3K: PI3 kinase
SNV: single nucleotide variant
WES: whole exome sequencing

## 1 Introduction

Treatment options that deliver long‐term control or eradication of disease are limited for many types of cancer. Often tumors do not respond to initial treatment or develop secondary resistance after initial response. There is a great need for continued anticancer drug development to continue to expand available treatment options. Natural products, which are derived from a variety of organisms, provide a diverse natural reservoir of chemical structures, which can potentially serve as novel sources for development into cancer therapeutics. Indeed, many natural products and compounds with structures that were based on natural product pharmacophores have been successfully utilized in the development of anticancer therapy agents that are now approved for clinical treatment or are currently in clinical trials [1, 2, 3, 4, 5]. Despite this success, a vast and diverse collection of natural compounds remains to be explored for their potential therapeutic properties. They may have utility as single agents, in combination with other therapies, as part of natural product‐antibody anticancer conjugates, or for the discovery of new drug pharmacophores [1, 2, 3].

The Natural Product Repository at the U.S. National Cancer Institute (NCI) has assembled a unique, extensive, and highly diverse collection of extracts of natural products isolated from various plant, marine, and microbial species from around the world [1, 6]. The Developmental Therapeutics Program (DTP) of the NCI has screened these compounds *in vitro* for their antitumor cytotoxic properties using the well‐characterized NCI‐60 cancer cell line panel [7, 8]. In addition, the DTP accepts outside submissions of chemical structures for the NCI‐60 screen, and together with natural products, this dataset is one of the largest collections of annotated small molecule cytotoxicity screens. In this study, a subset of 1302 small molecule structures, which included natural products, semisynthetic natural product derivatives, and synthetic compounds based on natural product pharmacophores isolated at the NCI or submitted by established natural product research groups were chosen for analysis. It includes well‐characterized natural products such as paclitaxel (NSC 125973), camptothecin (NSC 302991) and its derivatives, colchicine (NSC 9170) and its derivatives, and centaureidine (NSC 106969), as well as less studied compounds isolated from plants, marine invertebrates, and microbes, many of which remain under investigation.

Molecular features of the NCI‐60 cell lines have been extensively characterized, with gene expression, whole exome sequencing (WES), copy number variation, DNA methylation, miRNA expression, and proteomics data that have been made publicly available [9, 10, 11, 12, 13, 14, 15, 16, 17, 18]. While this wealth of molecular information has been successfully used in a variety of drug response studies of FDA‐approved and investigational oncology agents [18, 19, 20, 21, 22] and for a small number of selected natural products with strong antitumor activity [23, 24], molecular feature data for NCI‐60 cell lines have not yet been utilized for a systematic understanding of tumor response to a broad range of natural products. With objectives to better understand mechanisms of cytotoxic action of different natural compounds and to identify potential targets and explanations for variation in drug response, we sought to investigate how molecular features of the NCI‐60 cell lines may contribute to the response to natural products. We examined the associations of 1302 natural compounds screened in the NCI‐60 panel with genome‐wide transcript expression and with known clinically or biologically important single nucleotide variants (SNVs), in order to suggest potential mechanisms and molecular targets of these natural compounds.

## 2 Materials and methods

### 2.1 Molecular screen of natural products for drug response

Among the molecular screening NCI‐60 data generated by the DTP, 1302 compounds were selected for detailed evaluation of their association with molecular features. All products were screened *in vitro* at five concentrations for their antitumor cytotoxic properties using the NCI‐60 cancer cell line panel [7, 8]. Sample handling, preparation, and cell line testing for sensitivity to natural compounds followed standard NCI‐60 screening assay protocols, including the 2‐day incubation and the use of sulforhodamine B endpoint assay. Detailed description of the sample preparation procedures is available at the NCI Division of Cancer Treatment and Diagnosis DTP NCI‐60 Screening Methodology website (https://dtp.cancer.gov/discovery_development/nci‐60/default.htm). Concentrations of all pure compounds were measured in molar units.

Sensitivity of each NCI‐60 cell line to each compound was quantified by a GI50 value, which represents the concentration producing 50% growth inhibition that is derived from the five‐concentration screen of each compound at 48 h after incubation [7]. If a GI50 value could not be derived for a specific biological experiment, or was not available from the NCI‐60 screening program, such value was set as missing. Following the NCI‐60 screening and data processing procedures, any GI50 value falling outside of the testing range for a given cell line and natural product had been assigned a value equal to either the highest or lowest concentration tested [7]. All nonmissing GI50 values were used in analyses after log_10_‐transformation. We further refer to these transformed values as log(GI50). Median log(GI50) values were computed using experimentally derived nonmissing log(GI50) data for all biological replicates of each cell line‐natural compound pair. These median values of cell line response were used in all subsequent analyses including clustering of cell lines and compounds, and association analysis with molecular features of NCI‐60 cell lines.

Similarities of response patterns among NCI‐60 cell lines across natural compounds, and clusters of natural products with similar patterns of cell line sensitivity were identified using hierarchical clustering and heatmaps derived using the packages ape v. 5.3 [25] and gplots in the r environment (https://www.r‐project.org) v. 3.5.3. Clustering was performed using the ‘average’ (UPGMA) option of the hclust function based on Euclidian distances. For these analyses, missing log(GI50) data were omitted from the distance matrix computation using pairwise deletion. dendroscope v. 3.6.2 and 3.7.2 [26] was used for visualization of the tree clustering of both the NCI‐60 cell lines and of the 1302 natural products.

We also searched for subsets of cell lines and natural products with similar patterns using the biclustering plaid model algorithm of Turner *et al*. [27]. We used the biclust software package v. 2.0.2 with default parameters for the plaid model, that is, with row and column release probabilities set at 0.7 and the maximum number of layers at 20, and using five iterations to find the starting values and 10 iterations to find each layer [28]. The resulting biclusters were visualized using the drawHeatmap function of the biclust package. In order to perform biclustering, missing log(GI50) data were imputed, with default parameters, using the missforest package v. 1.4., which employs a random forest‐based iterative imputation algorithm [29]. Imputed log(GI50) values were used in the biclustering analysis only.

To investigate the potential mechanism of action of the 2‐phenyl‐4‐quinolones NSC 656161, which emerged as a significant hit in association analysis with gene expression, similarities between NCI‐60 cell line response to this natural product and response to previously approved or developmental antitumor agents were assessed *via* the online COMPARE portal at the NCI (https://dtp.cancer.gov/databases_tools/compare.htm) [8, 30], which uses Pearson correlation analysis of the GI50 values of the NCI‐60 cell lines to assess pairwise similarity between pairs of agents. We performed the search of the GI50 values for the NSC compound data using the COMPARE portal, with NSC 656161 used as a query input [31]. Agent matches identified by COMPARE with the absolute value of the Pearson correlation coefficient |*r*| > 0.4 were examined. To further assess and validate these hits and to analyze the patterns of the NCI‐60 cell line response, the GI50 values of antitumor agents identified by COMPARE as having similar response patterns to NSC 656161, and of temozolomide, which may have a similar mechanism of response to that of NSC 656161, were obtained from publicly available resources (June 2016 release) at the NCI (the NCI DTP databases and tools portal, https://dtp.cancer.gov/databases_tools/default.htm, and the NCI DTP bulk data for download: NCI‐60 growth inhibition data website, https://wiki.nci.nih.gov/display/NCIDTPdata/NCI‐60+Growth+Inhibition+Data). Patterns of similarity of log(GI50) response between candidate agents and NSC 656161 were analyzed using Pearson and Spearman correlation and scatterplots.

Publicly available chemical annotation of the natural compounds and data about their bioactivity were obtained from PubChem (https://pubchem.ncbi.nlm.nih.gov) and from the NCI DTP online portals for public bulk data download and online search (https://wiki.nci.nih.gov/display/NCIDTPdata/Chemical+Data and https://dtp.cancer.gov/databases_tools/default.htm). pubchem sketcher v.2.4 (https://pubchem.ncbi.nlm.nih.gov/edit3/index.html) and perkinelmer chemdraw professional, USA v. 18 software were used for visual representation of the chemical structures.

Chemical structures of the 77 compounds discussed in the text of this report are presented in Table S1. They were drawn using the perkinelmer chemdraw professional v. 18 software, based on the SMILES information obtained from the DTP Repository. In order to cluster these chemical structures, we utilized the chemical software chemminer [32] to generate the distance matrix among structures, using the Single‐linkage binning cmp.cluster command on an atom pair object. Clustering of the chemical structures was performed using the hclust function in r and visualized using dendroscope v. 3.7.2.

### 2.2 Associations between NCI‐60 response to natural products and gene expression

**Fig 1:**
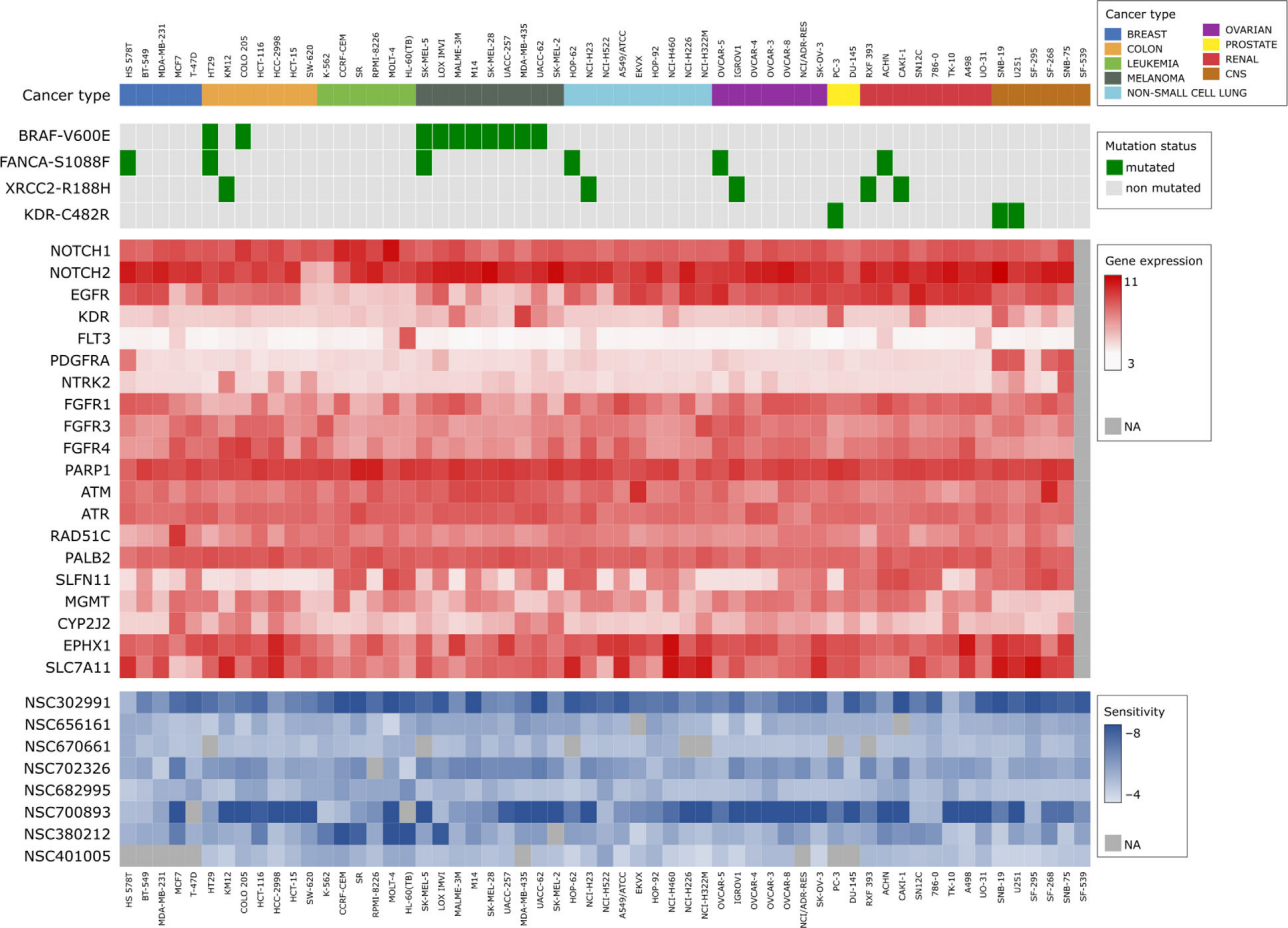
An overview of selected molecular features of NCI‐60 cell lines. Shown is the D3Oncoprint [38] diagram of the NCI‐60 cell lines used in analysis, indicating their cancer categories, selected genomic alterations and expression values for several biologically important genes. Also shown are sensitivity values of each cell line to selected natural products. Columns represent the cell lines, whereas rows represent SNV variants, gene expression values, and measures of sensitivity to natural products. The top panel depicts selected genomic alterations and the mutation status in each cell line. The center panel shows a heatmap of log_2_‐transformed gene expression values. The bottom panel shows the heatmap of the median log(GI50) values for selected natural products. Cell lines are ordered according to their tumor categories.

In order to examine potential mechanisms of the cytotoxicity of the selected natural products, we analyzed associations between the cell line sensitivity to these agents and molecular features of the NCI‐60 cell lines including their gene and miRNA expression, and the presence of potentially relevant SNVs. Gene expression microarray data for the NCI‐60 cell lines were downloaded from the CellminerCDB resource (https://discover.nci.nih.gov/cellminercdb/) [33]. NCI‐60 WES information was downloaded from the CellMiner data download site (https://discover.nci.nih.gov/cellminer/loadDownload.do) [34]. Figure 1 provides a graphic overview of the SNV status and gene expression measures for selected functionally important variants and genes in the NCI‐60 dataset. We analyzed associations of cell line molecular features with response to the 1291 out of 1302 natural products and compounds that had variation in their log(GI50) values among the NCI‐60 cell lines. The remaining 11 natural products and compounds did not have any variation in their response. Among the 61 NCI‐60 cell lines that were profiled in different molecular assays and/or for response to natural compounds, the MDA‐MB‐468 cell line did not have molecular data in CellMiner, and the MDA‐N cell line was not screened for response to the natural products. Both of these cell lines were excluded from our analysis of associations between log(GI50) and WES information of the NCI‐60 cell lines, which was based on 59 cell lines that had both molecular data and log(GI50) data. Analysis of correlations between log(GI50) and gene expression of the NCI‐60 cell lines further excluded the SF‐539 cell line which did not have expression data in CellMinerCDB, resulting in the total of 58 cell lines.

For transcriptional analysis, we used gene expression microarray data for the NCI‐60 cell lines. Their log_2_‐transformed expression levels for 23 059 gene and miRNA transcripts were downloaded from the CellMiner [34] using an average intensity format. These transcriptional measures had been generated using an integration of five gene expression microarray platforms (Affymetrix Human Genome HG‐U95, Affymetrix Human Genome HG‐U133, Affymetrix Human Genome U133 Plus 2.0, Affymetrix GeneChip Human Exon 1.0 ST (ThermoFisher Scientific, Santa Clara, CA, USA), and Agilent Whole Human Genome Oligo arrays (Santa Clara, CA, USA)). Normalization procedures for each array and integration of transcriptional measures were described previously [10, 35].

Correlation analyses were performed using r v. 3.5.3. Association between pretreatment log_2_‐transformed gene expression measures and log(GI50) of drug response was examined using Spearman correlation analysis. Cell line pairs with missing expression or log(GI50) values were excluded from correlation using pairwise deletion. The resulting *P*‐values were adjusted for multiple testing using the Benjamini‐Hochberg's procedure [36] to control the false discovery rate (FDR), while accounting for all 1291 natural products and all transcripts. We selected genes associated with response to natural products using the cutoff of *P* < 0.05 for FDR‐adjusted *P*‐values. When more stringent Bonferroni significance was achieved it was also noted. Information about the functional roles of genes associated with natural product expression was obtained from GeneCards (https://www.genecards.org/) [37].

In addition, we examined the significant associations between cell line response to natural products and expression of genes representing known therapeutic targets or genes reported to affect response to known antitumor agents with established mechanisms of action. These genes included *BRCA1*, *BRCA2*, *PALB2*, *PARP1*, *PARP2*, *PARP3*, *CHEK1*, *RAD51*, *RAD52*, *RAD51C*, *ATM*, *ATR*, *SLFN11*, *EGFR*, *KDR*, *FLT3*, *FLT4*, *KIT*, *SCR*, *PDGFRA*, *PDGFRB*, *NTRK1*, *NTRK2*, *FGFR1*, *FGFR2*, *FGFR3*, *FGFR4*, *AURKA*, *AURKB*, *AURKC*, *NOTCH1*, *NOTCH2*, *NOTCH3*, *NOTCH4*, and *MGMT*.

Molecular features of the NCI‐60 cell lines and their responses to natural products were visualized using the D3Oncoprint software developed by our group [38].

### 2.3 Analysis of associations between cell line response to natural products and the presence of NCI‐60 single nucleotide variants

In order to analyze DNA variants with known biological effects in cancer cells which may be associated with the cytotoxicity response to natural products, we compiled the list of genes with SNVs known to be likely clinically or biologically important in cancer development, progression, or drug response according to the OncoKB resource (http://oncokb.org) [39], which classifies genomic variants according to levels of clinical or biological evidence. A list of candidate genes and functionally relevant SNVs from OncoKB v. 1.17 was generated by including variants classified by OncoKB at levels 1–4 of potential therapeutic actions, R1 and R2 levels of resistance, and variants classified as ‘oncogenic’ and ‘likely oncogenic’ [39]. We used this list to examine the presence of important variants in the NCI‐60 WES data from the CellMiner online resource [34], which provides multiple molecular datasets for the NCI‐60 cell lines for download. We evaluated all protein‐changing SNVs recorded in CellMiner that occur in the genes derived from OncoKB using the levels 1–4, R1, R2, ‘oncogenic’, and ‘likely oncogenic’ criteria. Using CellMiner WES data, we identified 1586 protein‐changing SNVs in 280 genes across 59 cell lines. For these 1586 protein‐changing alterations, we used a filter, wherein both the group of the NCI‐60 cell lines that had a particular SNV variant and the group of cell lines that did not have that variant, each had at least three cell lines. We accounted separately for each individual variant, which resulted in 107 genes with 220 SNVs across 59 cell lines, with each SNV present in ≥ 3 cell lines. We further evaluated the frequency of each variant in the 1000 Genomes dataset [40] according to the information provided by the CellMiner. A flowchart demonstrating the filtering of NCI‐60 molecular data for DNA variants is summarized in Fig. S1.

To examine the associations of individual protein‐changing SNVs in each gene listed in OncoKB, we used Student's *t*‐test to compare the log(GI50) values between groups of cell lines having any protein‐changing variant in a given gene and those not having any protein‐changing variant in that gene. To account for missing response data of individual cell lines to specific natural products, we restricted our analysis to the SNV‐natural product pairs which had ≥ 3 cell lines without any missing data in variant status and log(GI50) in each comparison group. The *P*‐values were FDR adjusted, accounting for the 1291 natural products and 220 SNVs.

In a separate analysis of the *EPHX1* gene, which was not listed in OncoKB but emerged as a candidate in our expression analysis, we used the *t*‐test to examine whether the response to any natural product was associated with two *EPHX1* polymorphisms, Y113H and H139R, which have been reported to modestly influence EPHX1 activity [41, 42]. *EPHX1* variant status of the NCI‐60 cell lines was downloaded from CellMiner. The *P*‐values were FDR adjusted, accounting for the 1291 natural products and both polymorphisms.

Analysis of molecular associations between response to the natural products and sequence variants was performed using the rstudio (Boston, MA, USA) v. 1.0.153 and r 3.6.3. Biological interpretation of significant SNV associations was based on SNV annotation in OncoKB and ClinVar (https://www.ncbi.nlm.nih.gov/clinvar/) [43] and on published reports in biomedical literature.

## 3 Results

### 3.1 Hierarchical clustering of NCI‐60 cell lines and natural products based on the log(GI50) measures

**Fig 2:**
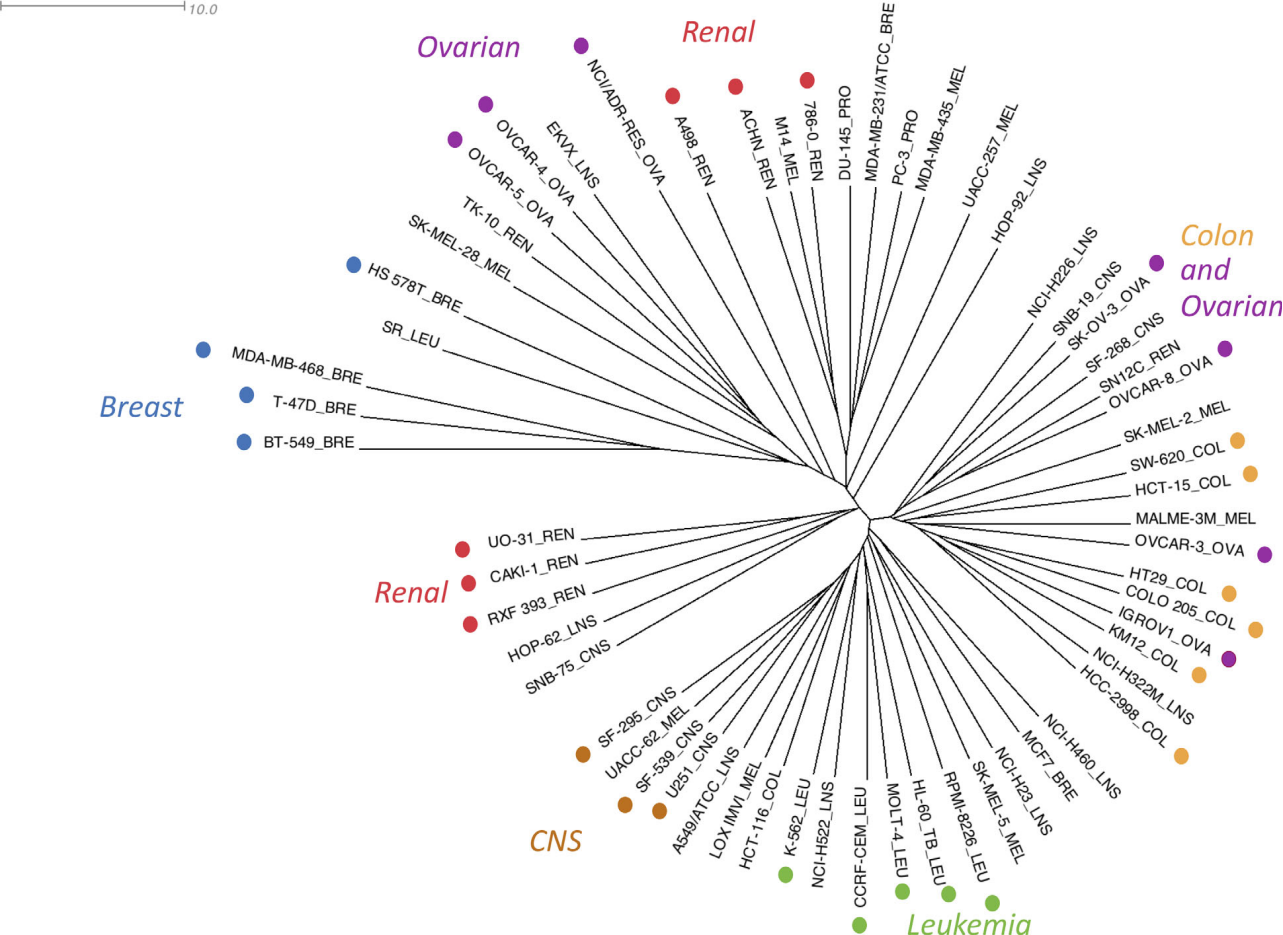
Hierarchical clustering of NCI‐60 cell lines based on the median log(GI50) values of 1302 natural products. The tree was inferred using the UPGMA (‘average’) method and was based on Euclidian distances. The tree is presented as an unrooted radial dendrogram [26, 44]. The scale in the top left corner is provided for the branch length, which were derived from Euclidian distances. Tumor category information is appended to cell line names. BRE, breast; COL, colorectal; LEU, leukemia; LNS, lung; MEL, melanoma; OVA, ovarian; PRO, prostate; REN, renal.

**Fig 3:**
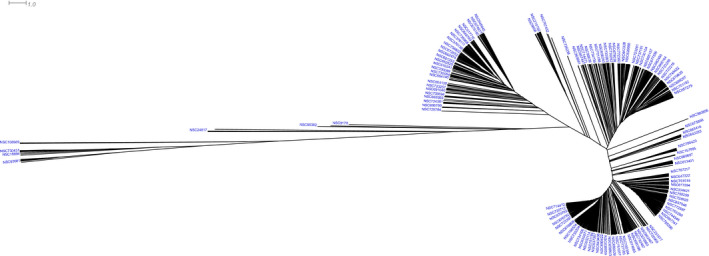
Hierarchical clustering of 1302 natural products based on the median log(GI50) values of 59 NCI‐60 cell lines. The tree is presented as an unrooted radial dendrogram [26, 44]. The tree was inferred using the UPGMA (‘average’) method and was based on Euclidian distances. The scale in the top left corner is provided for the branch length, which were derived from Euclidian distances. Only selected natural product names are shown. All 1302 natural product names can be viewed in Fig. S3.

Figure 2 shows hierarchical clustering of the NCI‐60 cell lines based on the median log(GI50) values, whereas Figure 3 provides an overview of hierarchical clustering of the natural products based on their median log(GI50) values. Both trees in Figs 2 and 3 are presented as unrooted radial dendrograms [26, 44]. Detailed clustering of the cell lines and of the natural products with full labeling is presented as midpoint rooted rectangular dendrograms [26, 44] in Figs S2 and S3. Figure S4 provides a heatmap showing the two‐dimensional clustering of the NCI‐60 cell lines and the natural products, in which similar groups among the 1302 natural products are clustered according to the similar patterns of cell line response to these compounds.

The unrooted tree [44] presented in Fig. 2 shows that, based on their response to the 1302 natural products, the NCI‐60 cancer cell lines were clustered in a star‐like manner, with long branches leading to each cell line and very short branches connecting clusters of cell lines. This suggests no clear separation of distinct cell line clusters based on cell line response profiles to all 1302 compounds. Some cell lines from leukemia, ovarian, renal, breast, and central nervous system (CNS) cancer categories, however, showed a tendency for clustering in the proximity of additional cell lines from the same histology (Fig. 2). In addition, one cell line cluster contained predominantly ovarian and colon cancer cell lines.

In contrast to the absence of distinct separation among the NCI‐60 cell lines, hierarchical clustering of the 1302 natural products resulted in their separation into distinct subgroups of natural compounds and their fractions (Fig. 3; Fig. S3). These subgroups were separated by long branches, suggesting that the patterns of cell line sensitivity to the compounds within specific clusters were considerably different from patterns of response to those from other clusters. The compounds within individual subgroups were clustered together when similar patterns of response to these compounds were observed across the NCI‐60 cell lines. Even though the clustering based on log(GI50) values did not utilize the information about molecular alterations of individual cell lines, similarities of response patterns within individual natural product clusters may suggest similar mechanisms of action among different compounds within specific clusters.

Interestingly, hierarchical and 2‐dimensional clustering (Fig. 3; Figs S3 and S4) suggested a distinct pattern of response for the hydroxy flavone centaureidin (NSC 106969) and deoxysterone diaziridinyl triazine analogue NSC 106909 ((8R,9S,13S,14S)‐3‐[[4,6‐Bis(aziridin‐1‐yl)‐1,3,5‐triazin‐2‐yl]oxy]‐13‐methyl‐7,8,9,11,12,14,15,16‐octahydro‐6H‐cyclopenta[a]phenanthren‐17‐one), both of which showed distinct patterns of sensitivity of specific cell line clusters which included several breast cancer cell lines (Fig. S4; these compounds are shown as the two top rows in the heatmap). Although the distinct clustering of these compounds could be potentially associated with the differences in the units of response measures for various compounds, both were measured in molar units, as were all other compounds in the dataset. While the results of the screening of NSC 106909 in mouse models have been inconclusive or showed the lack of activity according to PubChem, centaureidin, a hydroxy flavone which has been isolated from a number of plant species from the *Asteraceae* family [45], is known for its strong *in vitro* antimitotic and cytotoxic activity against cell lines from multiple tumor categories [46, 47, 48]. The pattern of response of NCI‐60 cell lines, which is similar between these structurally unrelated compounds but is distinct from other natural compounds, is of interest.

### 3.2 Biclustering analysis

Figure S5 shows six biclusters identified as separate layers using the plaid model. They include groups of cell lines and natural products with similar patterns of response [49]. Biclusters 1 and 2 included very large numbers of cell lines (60 and 41, respectively) indicating more global effects on cell line response [27]. Smaller biclusters (3 through 6) included subsets of cell lines and natural products, suggesting distinct subgroups with similar response. The cell line composition of biclusters 4 and 5 was similar to that of several hierarchical clusters of the NCI‐60 cell lines, which were identified based on response to all natural products (Fig. 2) and linked together. Bicluster 4 with 12 cell lines and four natural products (Fig. S5D) included breast, melanoma, ovarian, prostate, and leukemia cell lines from hierarchical clusters in the upper left corner of Fig. 2. The cell line composition of bicluster 4 was similar to that of bicluster 5. The latter included a number of the same cell lines (e.g., ovarian OVCAR‐4, OVCAR‐5, breast BT‐549 and TD‐47, and renal TK‐10; Fig. S5E) and additional cell lines (e.g., melanoma UACC‐257 and lung EKVX), which were also included in the same hierarchical clusters in Fig. 2. In contrast, bicluster 3 (Fig. S5C) with two natural products and 20 cell lines and bicluster 6 (Fig. S5F) with 11 natural products and 19 cell lines combined the above cell lines with several additional cell lines (e.g., melanoma SK‐MEL‐2 and MALME‐3M, ovarian IGROV‐1 and OVCAR‐3, and colorectal HT‐29, COL‐205, and KM‐12) from a separate hierarchical cluster in the right part of Fig. 2. Some distinct groups of natural products with diverse antitumor mechanisms and cytotoxicity [50, 51, 52, 53] were included in separate biclusters, for example, camptothecin derivatives in bicluster 6, fluorinated quinolones and 2‐aryl‐naphthyridin‐4‐ones in bicluster 5, and the plant oxoaporphine alkaloid liriodenine in bicluster 3.

### 3.3 Hierarchical clustering of chemical structures

Figure S6 presents hierarchical clustering of the chemical structures of 77 natural products from Table S1. Camptothecin analogs and its derivatives formed three distinct but related clusters based on their structural similarities. With a few exceptions, these clusters, which were based on structure similarities, were mostly similar to two separate hierarchical clusters of camptothecin and its derivatives which were identified from log(GI50) data (Fig. S3). The first cluster of structures included camptothecin (NSC 302991) and its analogs and derivatives, NSC 94600, 100880, 111533, 369395, 603071, 671901, 673591, 673592, 673593, and 673595 (Fig. S6), which were also clustered together based on log(GI50) data (Fig. S3). This cluster also included NSC 105132, 328410, 606173, and 673596, even though they were separated from other compounds from that cluster in the tree based on log(GI50) (Figs S3 and S6). NSC 616241 and 606985 formed a separate second cluster of structures, which was linked to the first cluster (Fig. S6). Both compounds were also clustered with camptothecin based on log(GI50) data (Fig. S3). The third cluster of camptothecin derivatives, which also formed a separate group in the log(GI50) tree, included NSC 681633 681634, 681635, 681637, 681639, 681640, 681642, 681644, and 681646. NSC 681643 was clustered with these structures (Fig. S6), even though it was separated from them in the log(GI50) tree (Fig. S3). The three clusters of structures of camptothecin and its derivatives were joined together and were separated from the remaining compounds (Fig. S6). In sections 3.4.1 and 3.5.2, we discuss the association of response to compounds from the three camptothecin clusters with *SLFN11* expression and those from the first cluster with the *PIK3CA* H1047R variant.

Among other compounds, chemically related triterpene glycosides NSC 676773, 676775, 676776, 676777, 676779, 676789, and 679973 formed a distinct cluster of structures (Fig. S6). They were also clustered together based on NCI‐60 response (Fig. S3). As discussed in section 3.4.6, NCI‐60 response to compounds from this cluster was associated with *PALB2* expression. The grouping of the structures of the majority of other compounds was less distinct, with long branches leading to individual compounds, reflecting the diversity of the structures (Fig. S6).

### 3.4 Association of cell line response to natural products with gene expression

Our correlation analysis between expression of 23 059 transcripts and median log(GI50) values of the 1291 natural products that exhibited variation of median log(GI50) values identified 24 465 natural product‐gene correlations that were statistically significant after adjusting for multiple testing (FDR adjusted *P* < 0.05). These significant results included 1134 natural products and 6320 transcripts (Table S2). Some of these correlations were very strong and highly significant. Statistically significant correlations for selected genes which are involved in specific pathways of drug sensitivity or drug action for a number of prespecified antitumor drugs are listed in Tables 1, Tables S3, S5, and S6. Below we discuss some significant correlations of a variety of biologically important genes and gene families.

**Table 1:** Selected statistically significant correlations of natural products and natural product fractions with expression of genes representing known drug targets. The *P*‐values reported in the table were adjusted for the FDR while accounting for the correlation tests involving all 1291 natural products and all 23 059 transcripts. The sample size represents the number of NCI‐60 cell lines with available gene expression and GI50 data for a given association. Sample sizes < 58 indicate missing data in some of the cell lines.

NSC	Gene	Spearman ρ	FDR adjusted *P*	Sample size	Agent
656161	*MGMT*	0.5859	0.0178	56	
656241	*NOTCH1*	0.5842	0.0184	56	Acetic acid, (4,8‐dioxobenzo[1,2‐b:5,4‐b']dithiophene‐ 2‐yl)methyl ester
676810	*NOTCH2*	0.5329	0.0363	58	Spiro[18‐norandrostane‐17,2'(3'H)‐furan]‐3,7‐diol, 3‐O‐acetyl‐7‐O‐(6‐O‐acetyl‐.beta.‐D‐glucopyranosyl)‐ 4',5'‐dihydro‐5'‐(1‐.alpha.‐hydroxy‐2‐methoxy‐ 2‐methylpropyl)‐3',4,4,8‐tetramethyl‐(3‐.alpha.,3'‐beta.,5‐.alpha.,7‐.alpha,17‐.beta.)‐
719985	*NOTCH2*	0.5385	0.0429	54	
682994	*PARP1*	−0.5509	0.0274	58	Benzo[1,2‐b:4,5‐b']dithiophene‐4,8‐diol, diester with butanoic acid
695403	*RAD51C*	−0.5249	0.0435	57	12‐Hydroxy‐bullatacin A
676793	*PALB2*	−0.5963	0.0137	57	
676775	*PALB2*	−0.5691	0.0204	58	Olean‐12‐en‐28‐oic acid, 3‐.beta.‐[2‐O‐(.beta.‐D‐glucopyranosyl)‐.beta.‐D‐glucopyranosyloxy]‐, [2‐O‐[[4‐O‐[3‐O‐(.beta.‐L‐arabinopyranosyl)‐.beta.‐D‐xylopyranosyl]‐3‐O‐.beta.‐D‐xylopyranosyl]‐ 6‐deoxy‐D‐gulopyranosyl]‐.beta.‐L‐arabinopyranosyl] ester
676776	*PALB2*	−0.5502	0.0277	58	Olean‐12‐en‐28‐oic acid, 3‐.beta.‐[3‐O‐(.beta.‐L‐arabinopyranosyl)‐.beta.‐D‐glucopyranuronosyloxy]‐, [2‐O‐[[4‐O‐[3‐O‐(.beta.‐D‐xylopyranosyl)‐.beta.‐D‐xylopyranosyl]‐3‐O‐(.beta.‐D‐xylopyranosyl)]‐ 6‐deoxy‐D‐gulopyranosyl]‐.beta.‐L‐arabinosyl] ester
676789	*PALB2*	−0.5400	0.0346	57	
676779	*PALB2*	−0.5307	0.0377	58	Olean‐12‐en‐28‐oic acid, 3‐.beta.‐[3‐O‐(.beta.‐L‐arabinopyranosyl)‐ 6‐methyl‐.beta.‐D‐glucopyranuronosyloxy]‐, [2‐O‐[[4‐O‐[3‐O‐(.beta.‐L‐arabinopyranosyl)‐.beta.‐D‐xylopyranosyl]‐3‐O‐(.beta.‐D‐xylopyranosyl)]‐ 6‐deoxy‐.beta.‐D‐gulopyranosyl]‐.beta.‐L‐arabinopyranosyl] ester
676777	*PALB2*	−0.5181	0.0453	58	Olean‐12‐en‐28‐oic acid, 3‐.beta.‐[3‐O‐(.beta.‐L‐arabinopyranosyl)‐ 6‐methyl‐.beta.‐D‐glucopyranuronosyloxy]‐, [2‐O‐[[4‐O‐[3‐O‐(.beta.‐D‐xylopyranosyl)‐.beta.‐D‐xylopyranosyl]‐3‐O‐(.beta.‐D‐xylopyranosyl)]‐ 6‐deoxy‐D‐gulopyranosyl]‐.beta.‐L‐arabinopyranosyl] ester
626165	*PALB2*	−0.5729	0.0491	45	Discorhabdin C‐benzene. TFA
676773	*PALB2*	−0.5114	0.0499	58	Olean‐12‐en‐28‐oic acid, 3‐.beta.‐[2‐O‐(.beta.‐D‐glucopyranosyl)‐.beta.‐D‐glucopyranosyloxy]‐, [2‐O‐[4‐O‐[3‐O‐(.beta.‐D‐xylopyranosyl)‐.beta.‐D‐xylopyranosyl]‐6‐deoxy‐.beta.‐D‐gulopyranosyl]‐.beta.‐L‐arabinopyranosyl] ester
682993	*ATM*	−0.5683	0.0207	58	Benzo[1,2‐b:4,5‐b']dithiophene‐4,8‐diol, dipropionate
690433	*ATM*	−0.5449	0.0303	58	Benzo[1,2‐b:5,4‐b']dithiophene‐4,8‐dione, 2,5‐bis(1‐hydroxyethyl)‐
682994	*ATM*	−0.5390	0.0331	58	Benzo[1,2‐b:4,5‐b']dithiophene‐4,8‐diol, diester with butanoic acid
656242	*ATR*	−0.5594	0.0274	56	

#### 3.4.1 *SLFN11*

The largest number of significant strong correlations with gene expression involved the *SLFN11* gene (Table S2 and S3). All significant correlations were negative (Spearman correlation coefficient ρ < −0.515, FDR adjusted *P* < 0.05; Table S3), indicating that elevated *SLFN11* expression was associated with sensitivity to these natural compounds. They included 27 highly significant associations with the *P‐*value prior to FDR adjustment between 8.74 × 10^−14^ and 7.98 × 10^−10^ (with Spearman ρ between −0.711 and −0.795) which satisfied the conservative Bonferroni threshold (8.40 × 10^−10^) for multiple testing of 1291 natural products with variable log(GI50) and 23 059 transcripts in a two‐sided correlation test. Increased *SLFN11* expression was previously associated with sensitivity to multiple categories of DNA‐damaging antitumor agents including topoisomerase I and II inhibitors, PARP inhibitors, and platinum compounds in a variety of cancers [54, 55, 56, 57, 58, 59, 60, 61, 62]. Figure S3 shows the midpoint rooted hierarchical clustering [44] of 1302 natural products based on their log(GI50) values across the NCI‐60 cell lines. Those compounds for which the sensitivity was significantly correlated with *SLFN11* expression (FDR adjusted *P* < 0.05) are highlighted in yellow. While all compounds were clustered based on similarities of their log(GI50) values, independent of cell line molecular features or cancer categories, this highlighting shows that levels of *SLFN11* expression were significantly associated with tumor cell line response to specific groups of natural compounds, which likely represent DNA‐damaging agents. Based on available annotation, many compounds associated with *SLFN11* expression may have topoisomerase I inhibition activity [24, 53, 63, 64]. For example, camptothecin (NSC302991), along with other topoisomerase I inhibitors, is well known for its association with *SLFN11* expression, including in the NCI‐60 panel [24, 53, 63, 64]. Other natural compounds in the same cluster with camptothecin (Fig. S3) include multiple camptothecin analogs and derivatives (e.g., NSC 606985, 94600, 100880, 369395, 111533, 681634, 681635, and 681643) [65], and a number of other compounds (e.g., NSC 616241, 671901, 673591, 673592, 673593, and 673595), suggesting possible topoisomerase I inhibition activity of the natural compounds in that cluster. Of note, a number of other camptothecin derivatives (e.g., NSC 105132, 673596, 328410, 606173, NSC 681633, 681637, 681639, 681640, 681642, 681644, 681646) were grouped in several clusters which were separate from the large cluster of camptothecin and its analogs, even though they were also significantly associated with *SLFN11* expression (Fig. S3; Table S3). This suggests that they may induce somewhat distinct patterns of response from those compounds in the large cell line cluster including camptothecin and its derivatives. NCI‐60 response to multiple quinoline derivatives, which are structurally related to camptothecin according to the information in PubChem, was also strongly associated with *SLFN11* expression (Table S3), in agreement with previously published data on noncamptothecin indenoisoquinoline derivatives, which also act as topoisomerase I inhibitors [53].

#### 3.4.2 *CYP2J2*

Expression of a cytochrome P450‐encoding gene, *CYP2J2*, was very strongly and significantly negatively correlated with log(GI50) of the cytotoxic antineoplastic agent austocystin D (NSC 700893; ρ = −0.792, *P‐*value prior to FDR adjustment = 3.75 × 10^−13^, FDR adjusted *P* = 1.86 × 10^−6^; Table S2), suggesting that cell lines with elevated expression of *CYP2J2* had an increased sensitivity to this product, which is produced by members of the fungal genus *Aspergillus*. This correlation, which satisfied the conservative Bonferroni threshold for significance, is in agreement with an earlier report of this association in an independent cancer cell line dataset from the Cancer Cell Line Encyclopedia and the Cancer Therapeutics Response Portal [66], and it is consistent with the action of austocystin D through selective activation of cytochrome P450 enzymes to invoke DNA damage in specific cell lines [67].

#### 3.4.3 *EPHX1* and *EPHX2*

Microsomal epoxide hydroxylase 1, the product of *EPHX1*, is involved in biotransformation of xenobiotic compounds, having dual roles in detoxification and bioactivation of a variety of substances. In particular, it plays an active role in the hydrolysis of epoxides derived from the degradation of aromatic compounds [41, 42]. In our dataset, increased expression of *EPHX1* was significantly associated with resistance to 8 compounds including anthraquinone derivatives NSC 380212, 673348, 673347, 673350; a bromopyrrole alkaloid NSC 626158, a quinone acridine NSC 694489, and other compounds (Table S2). Among the annotated natural compounds associated with expression of this gene were a CDK1/CDK2/GSK3β inhibitor bromopyrrole alkaloid called hymenialdisine (NSC 626158), isolated from a variety of marine sponges [68] and several aromatic compounds including 2‐[[4‐(oxiran‐2‐Ylmethoxy)naphthalen‐1‐Yl]oxymethyl]oxirane (NSC 673347), 2‐(6,11‐dioxobenzo[b]acridin‐12‐yl)prop‐2‐enal (NSC 694489), and two very strongly associated anthraquinones satisfying the stringent Bonferroni threshold, 1,4‐bis‐(2,3‐epoxypropylamino)‐9,10‐anthracenedione (NSC 380212, with reported antineoplastic activity [5]) and 9,10‐anthracenedione, 1‐[(oxiranylmethyl)amino]‐ (NSC 673348), with ρ = 0.766 and 0.741, *P* prior to the FDR adjustment = 3.90 × 10^−12^ and 3.05 × 10^−11^, FDR adjusted *P* = 9.68 × 10^−6^ and 4.79 × 10^−5^, respectively. These associations are consistent with the likely role of *EPHX1* in biodegradation of these natural products when its expression is elevated. Interestingly, expression of another epoxide hydroxylase gene family member, *EPHX2*, which encodes a soluble product with a more narrow substrate specificity for biotransformation activity [41, 42], was significantly associated with sensitivity, rather than resistance, to the meroterpenoid dihydroquinone natural product napyradiomycin A80915B (NSC 749271; ρ = −0.537, FDR adjusted *P* = 0.0340).

Based on the consideration that increased *EPHX1* expression was correlated with resistance to multiple natural products, we further investigated whether two common *EPHX1* polymorphisms, Y113H and H139R, were associated with response to any compounds. These variants have been reported to affect the biotransformation activity of EPHX1, although their effect is inconsistent and variable among previously reported compounds [41, 42]. In our dataset, the H139R variant, which was present in eight out of 59 NCI‐60 cell lines, had suggestive associations with sensitivity to 128 and resistance to 11 natural products (*P* between 2.44 × 10^−5^ and 0.0493 prior to FDR adjustment; Table S4), including sensitivity to an anthraquinone derivative NSC 673348 (*P* = 0.0075) and hymenialdisine NSC 626158 (*P* = 0.0125). Resistance to both compounds was significantly associated with increased *EPHX1* expression (Table S2). Other examples of H139R associations include sensitivity to NSC 735204 (5‐hydroxy‐7‐[3‐methoxy‐4‐(tetrahydro‐pyran‐2‐yloxy)‐phenyl]‐ 4‐{3‐[3‐methoxy‐4‐(tetrahydro‐pyran‐2‐yloxy)‐phenyl]‐acryloy l}‐hepta‐2,4,6‐trienoic acid ethyl ester; *P = *2.44 × 10^−5^ prior to FDR adjustment, FDR adjusted *P* = 0.0630), quinone‐containing pleurotin NSC 401005, several compounds that clustered with camptothecin derivatives (e.g., NSC 673591, 673593, 673595 and 105132, Fig. S3), the alkaloid discorhabdin C NSC 626162, and the diterpene jatrophone NSC 135037 (Table S4). Y113H, which was present in 28 cell lines, was associated with resistance to 44 and sensitivity to 20 products (Table S4; 0.0023 ≤ *P* ≤ 0.0494), including, for example, resistance to the quinolinedione derivative NSC 682995. Previous studies reported a modest reduction of EPHX1 biotransformation activity of certain compounds in the presence of these two variants [42]. While none of the associations of the natural products with these two variants achieved *P* < 0.05 after adjustment for the multiple testing of 1291 products in our data (Table S4), associations of *EPHX1* expression or polymorphisms with *in vitro* cell line response to treatment suggest that the presence of higher levels of functionally active EPHX1 in tumor cells may increase the tumor cell resistance to some natural products due to a more effective degradation of these compounds.

#### 3.4.4 *MGMT*

**Fig 4:**
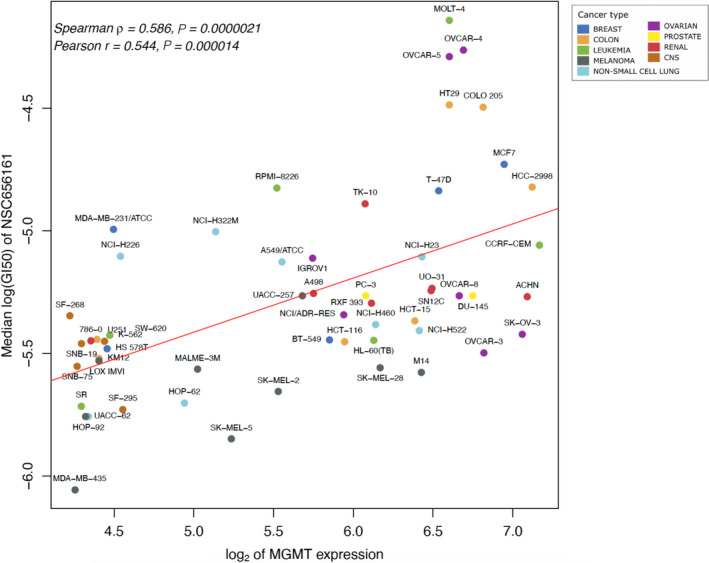
Scatterplot of median log(GI50) of the compound NSC 656161 vs log_2_‐transformed *MGMT* expression. The color representation of cancer categories is identical to that in Fig. 1.

We observed a significant correlation (Spearman ρ = 0.586, FDR adjusted *P* = 0.0178) between increased expression of *MGMT* and NCI‐60 cell line resistance to 2‐phenyl‐4‐quinolone analogue NSC 656161, annotated in PubChem as 6‐(3‐Bromophenyl)[1,3]dioxolo[4,5‐g]quinolin‐8‐ol (Fig. 4; Table 1). The cell lines that were sensitive to NSC 656161 and had low *MGMT* expression belong to a variety of tumor types including melanoma (e.g., MDA‐MB‐435, LOX IMVI, and UACC‐62) in which *MGMT* expression is typically low [69], and other cancers including brain (e.g., SF‐268, SF‐295, SNB‐19, SNB‐75), colorectal (SW‐620), leukemia (SR), nonsmall cell lung cancer (HOP‐92), and renal (786‐0; Fig. 4). The mechanism of action of NSC 656161 could resemble that of temozolomide (NSC 362856), due to the fact that elevated *MGMT* expression is a strong predictor of resistance to temozolomide in glioma and colorectal cancer [69, 70, 71]. MGMT counteracts the action of temozolomide by demethylating O^6^‐methylguanine (O^6^‐meG) lesions and removing larger O^6^‐alkyl adducts induced by temozolomide and nitrosourea‐based alkylating agents [72]. The chemical structure of the compound NSC 656161 (C_16_H_10_BrNO_3_) is distinct from that of temozolomide (C_6_H_6_N_6_O_2_; Fig. S7). Despite their different chemical structures, one could speculate that these two compounds could induce DNA lesions based on their negative association with *MGMT* gene expression. Though the NCI‐60 cell line response to temozolomide and NSC 656161 showed no correlation between their log(GI50) values (Spearman correlation coefficient ρ = −0.082, Pearson *r* = −0.085), the lack of correlation based on *in vitro* cell line response does not necessarily reflect any potential similarities *in vivo*, because temozolomide is a prodrug. Cytotoxic effects of temozolomide in the body occur after it is converted to an active compound [71]; therefore, its cell line sensitivity measures may not provide an accurate estimate of its efficacy or mechanism of action. Our search among the chemical agents screened by NCI DTP also identified PCNU (NSC 95466, 1‐(2‐Chloroethyl)‐3‐(2,6‐dioxo‐3‐piperidyl)‐1‐nitrosourea) as having a modest correlation with cell line response to NSC 656161 (Pearson *r* = 0.402 for similarity of its log(GI50) values to those of NSC 656161). PCNU is an alkylating nitrosourea compound [73], and the similarity between the *in vitro* response to that agent with that of NSC 656161 could also be influenced by *MGMT* expression levels in the NCI‐60 cell lines. Association of NSC 656161 response with *MGMT* expression and the similarity of its cell line response to PCNU further suggest that NSC 656161 could have a role in inducing DNA damage.

#### 3.4.5 *NOTCH* family

NCI‐60 response to the benzodithiophene derivative NSC 656241 (2‐[(2‐Benzotriazol‐1‐yl‐acetyl)‐(3‐ethoxy‐propyl)‐amino]‐N‐furan‐2‐ylmethyl‐2‐pyridin‐4‐yl‐acetamide, according to PubChem) was associated with *NOTCH1* expression, whereas *NOTCH2* was associated with two different compounds (Table 1). All correlations with *NOTCH* gene expression were positive, indicating that higher levels of expression of both *NOTCH* genes were associated with cell line resistance to these products. The Notch pathway plays important roles in oncogenicity, cell survival, and promoting and maintaining stem cell‐like and anti‐apoptotic properties [74]. Notch signaling has been associated with resistance to multiple chemotherapy agents and radiotherapy [74, 75]. Activation or overexpression of *NOTCH* family members was associated with endocrine resistance of breast tumor cells [76]. Many other potential resistance mechanisms are regulated by Notch signaling, for example, inhibition of apoptosis, miRNA regulation, direct activation of *HER2* expression, upregulation of the *ABCC1* (*MRP1*) transporter gene involved in multidrug resistance (MDR), and promotion of self‐renewal and stemness of tumor cells [74, 75, 77, 78]. Specific molecular mechanisms, which may explain associations between *NOTCH* family members and natural products in Table 1, will require further experimental investigation.

#### 3.4.6 DNA damage response pathway

Among the genes involved in DNA repair, no natural products were significantly associated with *RAD51*, *RAD52*, *PARP2*, *PARP3*, or *CHEK1* expression. While *BRCA1* expression can be silenced epigenetically in breast tumors [79], the expression of neither *BRCA1* nor *BRCA2*, both of which encode proteins involved in homologous recombination repair of double strand DNA breaks [80], was significantly associated with response to any of the 1302 natural products. *PALB2*, which interacts with both BRCA1 and BRCA2 in DNA damage repair, acts as a partner of BRCA2 in its stabilization, recruits it to the sites of DNA damage, and participates in its interactions with RAD51C [79, 81, 82], was associated with a group of chemically related (and similar both structurally and in their log(GI50) response, Figs S3 and S6) triterpene glycosides NSC 676793, 676775, 676776, 676789, 676779, 676777, and 676773 as well as the alkaloid discorhabdin C‐benzene NSC 626165 (Table 1). *RAD51C* (*FANCO*), a participant in DNA repair in the nucleus and mitochondria, and *PARP1*, which recruits DNA repair complexes to the location of single strand DNA breaks in the nucleus [80, 82, 83], were significantly associated with NCI‐60 response to the acetogenin natural product 12‐hydroxy‐bullatacin A (NSC 695403) and benzodithiophene‐4,8‐diol dibutanoate (NSC 682994), respectively (Table 1). Among the genes encoding homologous recombination repair kinases [82], a series of benzodithiophene diol and diene derivatives (NSC 682993, 690433, and 682994) were associated with *ATM* expression, whereas benzodithiophene dione NSC 656242 was associated with *ATR* expression. Among these products, NSC 682994, which is described in PubChem as benzo[1,5‐b']dithiophene‐4,8‐diol, diester with butanoic acid, was significantly associated both with *PARP1* and *ATM* expression (Table 1). Surprisingly, all significant associations with expression of *PALB2*, *RAD51C*, *PARP1*, *ATM*, and *ATR* with response to these compounds were negative, indicating that cell lines with higher levels of expression of these genes were more sensitive to associated structures. The direction of these correlations contrasts with known associations of the loss of expression or activity of several of these genes with sensitivity to DNA‐damaging agents [80, 81, 84, 85]. Therefore, the possible mechanism of action of these natural products in tumor cells, whereby increased levels of DNA repair are associated with increased sensitivity, requires further investigation. Increased sensitivity to NSC 690433 and NSC 682994, members of the benzodithiophene‐4,8‐dione group, was previously associated with increased chromosomal structural heterogeneity of NCI‐60 cell lines, a karyotypic measure of the number of nonclonal structurally rearranged chromosomes per metaphase [86] which could potentially indicate impaired DNA repair pathways; however, such measures of broad chromosomal instability could be induced by deficiencies in a broad range of DNA repair and maintenance factors and not limited to the genes found to be associated in our study [87, 88].

#### 3.4.7 Kinase genes

None of the compounds analyzed in this study were significantly associated with expression of kinase genes *FLT4*, *KIT*, *SRC*, *PDGFRB*, *NTRK1*, *FGFR2*, or Aurora kinase inhibitor genes *AURKA*, *AURKB*, or *AURKC*. Pretreatment expression levels of *EGFR*, *KDR*, *FLT3*, *PDGFRA*, *NTRK2*, *FGFR1*, *FGFR3*, and *FGFR4* were associated with cell line response to one or more natural products (Table S5).

*EGFR* had the largest number (51) of significant correlations; however, 50 of them were positive, suggesting that increased *EGFR* expression was associated with resistance to these natural products. Since some earlier reports, including our analysis of *in vitro* data, observed associations of *EGFR* overexpression with sensitivity to EGFR‐targeting agents erlotinib and gefitinib [9, 89, 90], whereas some clinical studies reported the absence of any association [91], the opposite direction of association with the natural products suggests that the primary mechanism of their action may not be targeting the EGFR pathway. Supporting this possibility, one of the associated agents, NSC665802 (seldane or terfenadine) is an antihistamine prodrug with *in vitro* antiproliferative and apoptotic activity through histamine receptor‐independent Mcl‐1 cleavage and Bak upregulation [92]. *EGFR* overexpression may indirectly influence resistance to natural products and involve additional mechanisms. For example, previous reports suggested its role in activating ATP‐binding cassette (ABC) family transporters involved in MDR [93], which could provide a potential explanation for some of the positive correlations observed in our data.

*FGFR1* was associated with three products, *FGFR4* with two products, and *FGFR3*, *FLT3*, *KDR, NTRK2*, and *PDGFRA* were associated with one product each. Interestingly, we observed correlations of *FGFR4* expression with log(GI50) of pomolic acid (NSC 670661; ρ = 0.576, FDR adjusted *P* = 0.0329; Table S5). Pomolic acid, an active triterpene from *Euscaphis japonica* and *Salvia officinalis* (sage), has strong antitumor and antiproliferative properties. It inhibits tumor cell growth, promotes apoptosis and can interact with or downregulate components of multiple cancer pathways [23, 94, 95]. Earlier studies of the NCI‐60 dataset suggested inhibition of the pathways in which EGF and EGFR are involved by pomolic acid [23]. The correlation between pomolic acid and *EGFR* expression in our dataset was very weak and did not reach statistical significance (ρ = 0.202, *P = *0.156 prior to FDR adjustment), in agreement with its previously reported lack of association with *EGFR* copy number, gene and protein expression, and mutation status [23]. The correlation of cancer cell sensitivity to pomolic acid with *FGFR4* expression observed in our study is notable and may provide better understanding of the mechanisms of action of this natural product and its potential use in combination treatment.

#### 3.4.8 Transporter genes

Table S6 provides the list of significant associations between log(GI50) of a variety of natural compounds and the expression of MDR *ABC* family transporter genes, which are involved in transport of a large variety of substances, including cancer drugs, across the plasma membrane and intracellular membranes [96, 97]. The genes involved in significant associations include *ABCB1* (ABC transporter‐subfamily B member 1, P‐gp, or MDR1), *ABCA12*, *ABCA3*, *ABCA5*, *ABCA6*, *ABCB5*, *ABCB7*, *ABCC1*, *ABCC2*, *ABCC3*, *ABCC4*, *ABCD1*, *ABCD2*, *ABCD3*, *ABCF2*, and *ABCG4* genes, along with two ABC transporter pseudogenes, *ABCD1P2* and *ABCF2P1*. The involvement of many transporter genes from the *ABC* family in chemoresistance to multiple agents has been extensively documented [96, 97, 98, 99, 100]. The associations found in this study demonstrate that many ABC family genes are also associated with cell line response to multiple natural compounds. Notably, we observed associations between expression of individual *ABC* family genes not only with chemoresistance, but also with chemosensitivity, which may be consistent with the reported roles of several natural products (tanshinone, tetrandrine, quercetin, grape‐seed polyphenols, tea polyphenol, curcumin, and baicalein) in reversing drug resistance by regulating the action of specific ABC transporters [100]. While the functional relevance of the two ABC transporter pseudogenes, *ABCD1P2* and *ABCF2P1*, is unclear, several *ABC* pseudogenes are co‐regulated and/or transcriptionally interfere with expression of the functional *ABC* family member genes [101], which provides a likely explanation for the association of these two pseudogenes with response to specific natural products.

Multiple genes from the solute carrier (SLC) families of transporters were associated with response to natural products. Among these genes were *SLC10A7*, *SLC11A1*, *SLC12A4*, *SLC15A2*, *SLC16A10*, *SLC16A12*, *SLC16A2*, *SLC16A5*, *SLC17A5*, *SLC17A7*, *SLC17A9*, *SLC18B1*, *SLC19A2*, *SLC19A3, SLC1A4, SLC22A10, SLC22A18, SLC22A23, SLC22A25, SLC22A3, SLC22A4, SLC23A1, SLC23A2, SLC25A11, SLC25A12, SLC25A15, SLC25A17, SLC25A19, SLC25A20, SLC25A25, SLC25A36, SLC25A4, SLC25A40, SLC25A5, SLC25A51, SLC26A10, SLC26A11, SLC26A8, SLC26A9, SLC27A1, SLC27A2, SLC27A3, SLC27A5, SLC29A1, SLC2A1, SLC2A10, SLC2A14, SLC2A3, SLC2A6, SLC2A8, SLC35B4, SLC35C2, SLC35F3, SLC35F5, SLC35F6, SLC35G1, SLC36A1, SLC36A4, SLC37A1, SLC37A3, SLC38A1, SLC38A2, SLC38A3, SLC38A5, SLC38A7, SLC38A9, SLC39A11, SLC39A13, SLC39A14, SLC39A3, SLC3A1, SLC41A2, SLC43A3, SLC44A3, SLC44A4, SLC48A1, SLC4A11, SLC4A3, SLC4A5, SLC4A8, SLC4A9, SLC50A1, SLC5A6, SLC5A8, SLC6A4, SLC7A1, SLC7A11, SLCO2B1,* and *SLCO5A1*), along *SLC9A3R1* and *SLC2A4RG* which are involved in the regulation or activity, localization, or transcription of *SLC* transporter genes according to GeneCards. In particular, increased expression of the cysteine‐glutamate transporter gene *SLC7A11,* which has previously been shown to be associated with response to a large number of antitumor agents including the antibiotic geldanamycin [102, 103], was associated with increased resistance to 42 natural products (Table S2) including a very strong (ρ = 0.763, FDR adjusted *P* = 0.00017) association with the naphthoquinone antibiotic pleurotin (NSC 401005; geogenine, or antibiotic P1), an anticancer inhibitor of thioredoxin reductase derived from the fungal species of *Pleurotus griseus*, *Geopetalum geogerium*, and *Hohenbuehelia* spp. [104, 105].

#### 3.4.9 *GPC1* and other glypican genes

We observed 56 significant associations (ρ between 0.513 and 0.713, FDR adjusted *P* between 0.00026 and 0.0490; Table S2) between expression of *GPC1,* which encodes the proteoglycan glypican‐1, and the response to a variety of natural products. All correlations were positive, indicating that increased expression of *GPC1* was associated with resistance to natural products. Two of the associations, with NSC 135037 (jatrophone, a macrocyclic diterpenoid with *in vitro* antitumor activity from the plant species from the *Jatropha* genus [106]) and NSC 626162 (discorhabdin C, a marine alkaloid derived from *Latrunculia* species sponges [107]), were highly significant and satisfied the conservative Bonferroni threshold (ρ = 0.713 with FDR adjusted *P* = 0.00026 for both agents, *P* prior to FDR adjustment = 3.33 × 10^−10^ and 3.44 × 10^−10^, respectively). In pancreatic cancer, glypican‐1 plays a role in the mitogenic response of malignant cells to the fibroblast growth factor FGF2 and the heparin‐binding epidermal growth factor HB‐EGF by forming ligand‐receptor complexes [108]. It is possible that increased *GPC1* expression could increase the mitogenic proliferation of the NCI‐60 cells, diminishing the cell line response to a number of natural products. Similar to *GPC1,* increased expression of *GPC6* was associated with resistance (Table S2). Interestingly, increased expression of *GPC3* was associated with sensitivity to NSC 664171 (4(1H)‐Quinolinone, 6‐(1‐pyrrolidinyl)‐2‐(3‐ methoxyphenyl)‐, a compound with potential tubulin‐binding properties according to PubChem; ρ = −0.518, FDR adjusted *P* = 0.0483; Table S2). While its product, glypican 3, is a prognostic and predictive marker and a therapeutic target in hepatocellular carcinoma [109], it may also modulate cell growth in epithelial cells, and the loss of the *GPC3* gene causes a congenital overgrowth syndrome with an increased risk of malignancies [108, 110]. It is possible that association of increased *GPC3* expression with sensitivity to NSC 664171 may involve growth modulating properties of its protein.

#### 3.4.10 *ELF* gene members of the epithelium‐specific ETS transcription factor gene family

The *ELF3* gene of the epithelium‐specific ETS transcription factor gene family family was significantly associated with 17 natural products including multiple dithiophene compounds (ρ between 0.514 and 0.711, FDR adjusted *P* between 0.00028 and 0.0478; Table S2). Increased *ELF* gene expression was associated with resistance to these compounds. Two significant associations, with NSC 682993 (Benzo[1,2‐b:4,5 b']dithiophene‐4,8‐diol, dipropionate) and NSC 690433 (Benzo[1,2‐b:5,4‐b']dithiophene 4,8‐dione, 2,5‐bis(1‐hydroxyethyl)‐), satisfied the Bonferroni threshold (ρ = 0.711, FDR adjusted *P* = 0.00028; *P* prior to FDR adjustment = 3.88 × 10^−10^ and 4.05 × 10^−10^, respectively). The role of dithiophenes in blocking the activity of another ETS factor, ERG, has been described previously [111, 112], suggesting a possibility that ELF3 may play a functional role in resistance to these compounds. In contrast to *ELF3,* the increased expression of the *ELF2* gene had only one significant association with increased sensitivity to NSC 678159 (ρ = −0.554, FDR adjusted *P* = 0.0252), whereas the elevated expression of *ELF1* had significant correlations with sensitivity and resistance to four various compounds (Table S2). Examples of compounds associated with sensitivity to *ELF1* include phenyl quinolone NSC 657278 and a madecassic acid derivative NSC 787217 (ρ = −0.556 and −0.532, FDR adjusted *P* = 0.0253 and 0.0445, respectively).

#### 3.4.11 Other associations

We observed significant associations of the *METTL7B* gene with colchicine (NSC9170) and of the *DOHH* gene with the hydroxy flavone centaureidin (NSC106969; Table S2). As discussed above, based on the hierarchical clustering of the natural products, the response of the NCI‐60 cell lines to centaureidin was very distinct from many other natural products. The biological implications of these associations require further experimentation.

### 3.5 Association of cell line response to natural products with protein‐changing single nucleotide variants

When using the Student's *t*‐test to analyze the differences between NCI‐60 cell line response to 1291 natural products with and without 220 individual protein‐changing SNVs from 107 genes listed in OncoKB, 3541 SNV‐compound pairs showed significant association (FDR adjusted *P* < 0.05) of differences in log(GI50) with the presence or absence of a given variant (Tables S7 and S8). While interpreting the associations of gene variants with drug sensitivity, we focused on 158 significant associations with exact matches to known deleterious variants in OncoKB at the individual variant level (Table S7), as the mechanism of action is best understood for such variants. Below we discuss selected examples of such associations.

#### 3.5.1 *BRAF* V600E

The *BRAF* V600E oncogenic variant has the strongest (1) clinical level of evidence in OncoKB. In the NCI‐60 dataset, it was present in most melanoma and two colon cancer cell lines (Fig. 1). This variant is clinically associated with response to BRAF inhibitors and BRAF‐MEK inhibitor combinations in melanoma according to OncoKB. In *BRAF* V600E‐positive colorectal tumors, which have different clinical response from melanoma [113], this variant is associated with clinical response to the BRAF inhibitor encorafenib in combination with immunotherapy agents, according to OncoKB. In our analysis, the presence of the *BRAF* V600E variant was associated with increased sensitivity to 30 natural products (Table S7). Among these compounds isobatzelline A NSC 682277 was highly significant (*P‐*value prior to FDR adjustment = 8.65 × 10^−14^, satisfying the stringent Bonferroni threshold; FDR adjusted *P* = 6.94 × 10^−10^) and exhibited mean difference in log(GI50) of 0.809 (−6.922 in the cell lines without the *BRAF* V600E variant and −7.731 with; Table S7). Among other examples of significant *BRAF* V600E associations with sensitivity to natural compounds, the tetracyclic plant triterpenoid cucurbitacin D (NSC 308606) with reported antitumor activity [114, 115] and the alkaloid isobatzelline D (NSC 682278) had FDR adjusted *P* = 6.94 × 10^−10^ and 0.0003, respectively. These compounds may be of interest in targeting cancers with *BRAF* mutations, and possibly act as BRAF inhibitors. Due to the presence of the *BRAF* V600E variant in eight out of nine melanoma cell lines in the NCI‐60 dataset, any association of *BRAF* V600E with specific natural products may indicate either the functional involvement of this variant or increased cancer‐specific sensitivity of melanoma cell lines to these compounds.

#### 3.5.2 Likely oncogenic variants

Multiple natural products were significantly associated with variants, which were annotated in the OncoKB as oncogenic or likely oncogenic, in the *FANCA, KDR, KIT, KNSTRN, MET, PIK3CA,* and *XRCC2* genes (Table S7).

Both FANCA and XRCC2 are involved in DNA repair pathways. In the NCI‐60 dataset, their likely oncogenic mutations were distributed across different cancers (Fig. 1). *FANCA* is a tumor suppressor gene and is a member of the Fanconi anemia pathway [116]. The likely oncogenic *FANCA* S1088F variant increases sensitivity to DNA‐damaging agents including cisplatin and mitomycin C [116]. Conversely, the likely oncogenic and likely loss‐of‐function *XRCC2* R188H variant increases resistance to cisplatin‐induced DNA damage [117]. Both SNVs were associated with sensitivity to the tubulin‐interacting natural product NSC 609394 (the macrolide homohalichondrin B) isolated from marine sponges [118]. Directions of associations of the log(GI50) of NSC 405647 (gelseminic acid) and NSC 106909, deoxysterone diaziridinyl triazine analogue (Estra‐1,5[10]‐trien‐17‐one, 3‐[[4,6‐bis(1‐aziridinyl)‐s‐triazin‐2‐yl]oxy]‐) with *XRCC2* R188H may suggest a possible DNA‐damaging effect of these natural products as it is similar to the associations reported for DNA‐damaging agents [116, 117]. NSC 405647, scopoletin (gelseminic acid, chrysatropic acid, 6‐methoxy‐7‐hydroxycoumarin), derived from the plant *Erycibe obtusifolia* Benth and other plant species, is a reported inhibitor of acetylcholinesterase which has antitumor, anti‐angiogenic, and anti‐inflammatory activities [119]. Importantly, the scopoletin derivative SC‐III3 directly induces DNA damage and causes activation of DNA damage‐related signaling pathways in malignant cells [119], which suggests that naturally‐derived scopoletin may also be involved in DNA damage as indicated by its association with the *XRCC2* R188H variant.

We also observed associations of the likely oncogenic, likely gain‐of‐function *KDR* C482R variant, according to OncoKB, with sensitivity and resistance to different natural products (Table S7). KDR is directly inhibited by VEGFR2 inhibitors, but it can also be inhibited by other tyrosine kinase inhibitors which have a broader range of targets [120]. The *KDR* C482R variant had been previously associated with decreased clinical response to pazopanib, a tyrosine kinase inhibitor [121]. In the NCI‐60 dataset, the resistance to NSC 682995 (2‐methyl‐5,8‐dihydro‐5,8‐dioxoquinoline, according to PubChem) was higher among cell lines with this variant (Table S7). It is possible that this natural product could have some tyrosine kinase inhibitor activity.

The likely oncogenic variant M541L in *KIT,* which encodes a tyrosine kinase receptor, was associated with sensitivity to the alkaloid bengamide B, NSC 646846, which had been initially isolated from a marine sponge, *Jaspis cf. coriacea* [122, 123]. Methionine aminopeptidases and NF‐κB were identified as molecular targets for bengamides [122, 124]. A new association of this compound with a mutation in a tyrosine kinase receptor may suggest a potential additional mechanism of its activity. Both *KIT* M541L and *FANCA* S1088F were also associated with sensitivity to the cactus alkaloid pilocereine NSC 21075 [125].

The T992I variant in the receptor tyrosine kinase *MET* gene was associated with sensitivity to halomon (NSC 650893), a halogenated monoterpene from the marine red algae *Portieria hornemanii* [126], NSC 644945, which had been reported to contain structural components of etoposide [127], and the podophyllotoxin analogue NSC 628676. It was also associated with resistance to discorhabdin I (NSC 656206), a cytotoxic alkaloid from *Latrunculia* species marine sponges [128].

The oncogenic driver mutation H1047R in the *PIK3CA* gene encoding the p110α catalytic subunit of PI3 kinase (PI3K) [129] was associated with sensitivity to NSC 679036 (1,8‐naphthyridin‐4(1H)‐one, 2‐(3‐chlorophenyl)‐6‐methyl‐), a cytotoxic compound inhibiting tubulin polymerization [130] and several camptothecin derivatives, for example, 9‐amino camptothecin NSC 603071 [131] and 369395 (Table S7; Figs S3 and S6). Sensitivity to 7‐ethyl‐10‐hydroxycamptothecin NSC 673596 was associated both with *PIK3CA* H1047R and *MET* T992I. These results are consistent with previously reported associations between activation of the PI3K/AKT pathway and the effect of camptothecin on tumor cells [132, 133]. NSC 603071, 369395, and 673596 clustered together based on their log(GI50) response and their structural similarities (Figs S3 and S6).

Among examples of other compounds associated with multiple OncoKB variants, sensitivity to colchicine, NSC 9170, was associated with *XRCC2* R188H, *KDR* C482R, and *KIT* M541L. *MET* T992I, *XRCC2* R188H, and the likely oncogenic variant A40E in the kinetochore gene *KNSTRN* were associated with resistance to centaureidine, NSC 106969 [134].

A number of protein‐changing SNVs in biologically and/or clinically important genes from OncoKB, which did not exactly match the OncoKB list of variants, also showed statistically significant associations with a variety of natural products (Table S8). While a number of these additional variants in the OncoKB genes are more likely to be benign, some of these SNVs may affect the biological function. Further investigation is needed to examine which of these associations may represent functional biological mechanisms of sensitivity or resistance, as opposed to spurious associations given the large number of associations examined.

## 4 Discussion

We analyzed associations of cancer cell line response to 1302 natural products and analogues with gene expression measures and SNVs in cancer‐related genes using data from the cancer cell lines in the NCI‐60 panel that has been used to test the anticancer activity of submitted compounds for over 30 years [7, 8]. Our findings confirmed some previously known information about the involvement of certain molecular factors in the tumor cell response to agents with specific chemical properties. For example, we confirmed the well‐established association between increased levels of *SLFN11* expression and sensitivity to camptothecin and derivatives thereof [24, 53, 63, 64]. We also confirmed the association of higher expression levels of *CYP2J2* with sensitivity to austocystin D [66, 67]. Moreover, we confirmed previously reported associations of many members of the *ABC* and *SLC* transporter families with response to treatment by multiple compounds [96, 97, 98, 99, 100] (Tables S1, S2, and S4), which are likely substrates for these efflux pumps.

Multiple potentially important novel associations were also identified, and these may provide new insights into molecular factors potentially involved in tumor chemoresistance and suggest possible mechanisms of action through which the cytotoxicity of some natural products is realized. For example, expression of *EPHX1* emerged in our study as a putative factor in tumor cell resistance to aromatic compounds (Table S2). We also observed an intriguing association between *MGMT* expression levels and response to NSC 656161, 6‐(3‐Bromophenyl)[1,3]dioxolo[4,5‐g]quinolin‐8‐ol (Fig. 4; Table 1), which may suggest similarities between the DNA‐damaging action of this compound and that of PCNU and possibly temozolomide.

Our analysis of response patterns of NSC 656161 and their comparison to PCNU and temozolomide supplements the results of previous studies which used NCI COMPARE to examine the potential mechanisms of action of various natural products, their derivatives, or compounds with similar structures [135, 136, 137, 138]. Many natural products previously examined by COMPARE or closely related natural compounds were included our study. For example, earlier studies employed this tool to evaluate similarities among known antimitotic agents and identified novel natural products with antimitotic properties in studies of paclitaxel, colchicine, halichondrin B, homohalichondrin B, dolastatin 10, combretastatin A‐4, and other natural products or their derivatives [139, 140]. COMPARE analysis also established unique response profiles of several diverse marine natural products including cephalostatins, halomon, and bengamide B [126, 139, 141, 142]. COMPARE was also used to search for topoisomerase I inhibitors among natural extracts with profiles similar to camptothecin and to extract camptothecin and 9‐methoxycamptothecin from a new plant source [143], and to investigate association of gene expression with response to pomolic acid [23].

Our clustering of 1302 natural products indicated that patterns of cell line response to some groups of compounds may be influenced by specific molecular factors, as demonstrated by the effect of SLFN11 on response to quinoline derivatives (Fig. S3). Those compounds, the sensitivity to which was significantly correlated *SLFN11* expression, were clustered together based on their log(GI50) values. Such associations may suggest specific molecular mechanisms of *in vitro* antitumor action of individual natural products for which such mechanisms have not yet been established. This would indicate that the natural compounds clustered closely together likely elicit similar responses of cell line sensitivity or resistance.

We focused on individual SNV variants with clearly defined mechanisms of sensitivity or resistance or pathogenic effects and analyzed associations of one molecular feature‐compound pair at a time. Further molecular studies may provide additional information about molecular features which could explain the grouping of the NCI‐60 cell lines based on their response to the 1302 natural products. In addition, investigation of underlying molecular causes of expression variation of individual genes associated with response to natural products, for example, epigenetic regulation, gene amplification or deletion, and gene fusions, may provide additional insights into tumor cell response to these compounds. Future studies could employ multivariable models using manually curated genomic alterations from multiple genes including specific SNVs, gene expression, fusions, and high level copy number alterations with known effects on drug response. Such multivariable analyses could be used both to search for natural products with novel antitumor mechanisms and to identify the natural products which may have similar molecular targets to known antitumor drugs but could potentially avoid known resistance mechanisms.

Our results provide a new insight about mechanisms of sensitivity and resistance to natural products which were associated with molecular genomic features of tumor cells. Phenotypic screening, including screening of natural products and compounds derived from natural substances, has been successful in tumor drug discovery as an alternative or complementary approach to molecular target‐based screening [144, 145, 146]. Future studies may extend an application of our approach to phenotypic screening of natural products, by focusing on the mechanisms of their action. For example, mining of genomic data, similar to the approaches successfully employed in phenotypic screens of small molecules [147], could be used to distinguish among multiple potential mechanisms which may affect an outcome of interest (e.g., cytotoxicity, cell morphology, stemness, or other properties). In addition to the data mining approach to identify the genetic components that could affect a specific phenotypic outcome (e.g., apoptosis, an activation of an efflux pump, reduced cellular uptake, or an alteration of a molecular target could all affect cytotoxicity) [148], knowledge of molecular information may allow the stratification of the cell lines or other screening models based on their molecular background, prior to the phenotypic screening of the natural products, which could potentially reduce the amount of the screening effort [149].

We analyzed pretreatment gene expression measures and sequence variants collected in nontreated tumor cell lines, and therefore any molecular features identified in our study as associated with cell line resistance to natural products were not acquired as the result of cell treatment with the natural products themselves. We cannot rule out the possibility that prior exposure of a tumor to a drug with similar mechanism of action to the natural product is responsible for the observed resistance. In the absence of treatment history, we cannot distinguish between intrinsic tumor characteristics and those acquired through epigenetic modifications or emergence of resistance mutations under pressure of prior therapy. However, since the NCI‐60 cell lines were derived from tumors several decades ago [7, 8] when older treatment options were available, it is reasonable to suggest that many of the associated molecular features in our study represent intrinsic tumor properties that were not acquired during treatment with similar compounds.

We used data from the NCI‐60 cancer panel because it provides a rich collection of molecular data and response measures for a large number of natural products, as well as reference measures for established drugs and investigational agents. The strengths (e.g., reproducibility, large amount of available screening and molecular data, and the use of standardized procedures) of this panel, its historic role in drug discovery, and its limitations (e.g., adaptation of cell lines to two‐dimensional culture and limited sampling of molecular alterations) have been discussed in detail [7, 8, 9, 131, 139, 150, 151]. The NCI‐60 panel is comprised of multiple tumor categories which are represented by a small number of cell lines. We were able to make our inferences based on the patterns of response to natural products, which could be observed across multiple cancer categories. This inference is consistent with earlier studies of established cancer drugs which suggested the utility of the NCI‐60 panel in pan‐cancer findings [20]. Our analysis represents an important initial step in evaluation of molecular associations with natural products. The effects of individual natural products or their synthetic analogs in specific cancer histologies could be investigated further using large sample sets of cell lines, organoids, or patient‐derived xenografts derived from a particular tumor category. Such complementary approaches of pan‐cancer analysis and analysis within specific cancer types may be beneficial for elucidating mechanisms of tumor sensitivity and resistance to natural products and for identifying promising products for pursuit in antitumor drug development.

In the absence of noncancerous cells in the NCI‐60 panel that could be used for comparison, the inferences made in our study about the effects of the natural products apply to malignant cells. Additional studies would be needed to evaluate the toxicity of the products studied in the noncancerous cells. Some of the associations with response to natural products identified in our study may arise due to somatic and epigenetic changes in the tumors and transcriptional dysregulation of malignant cells. Some other variants, including common *EPHX1* polymorphisms and some oncogenic and likely oncogenic SNVs from OncoKB, may represent germ line variants that could also be present in the noncancerous tissues of the cancer patients. Similarly, interindividual and tissue‐specific expression of some of the genes identified in our study could also vary in normal tissues. Therefore, a follow‐up investigation of the effects of specific natural products in normal tissues with different molecular backgrounds may be important.

We used a single phenotypic variable, log(GI50), as an outcome measure. Such single value may not capture the complexity of the tumor cell response at different concentrations of natural products. If the activity of a natural product fell outside the screening range, the use of the log(GI50) values assigned at the boundary of the range of the screening concentrations likely resulted in some loss of information about tumor response. For specific products and associations of interest, additional screens could be pursued at an expanded range of screening concentrations, accompanied by functional *in vitro* studies. Such follow‐up experimental analyses could refine the and validate the biological nature of observed associations of individual natural products with molecular features of tumor cells.

Our analysis involved the *in vitro* screen of 1302 natural products and analogues including well‐studied compounds and some less investigated pure compounds. Although the use of cell lines has some limitations by not allowing the investigation of clinically relevant physiological or immunological response to the screened compounds [4], our results demonstrate the benefits of identifying molecular features of tumor cells which are associated with sensitivity or resistance to individual natural products. Such findings provide a plausible way of elucidating the potential mechanisms of action of natural products within tumor cells and may suggest hypotheses for further pursuit in drug discovery studies. In addition, the methodology utilized in this study may enable broader research studies with natural product extracts and fractions. We are currently applying a similar analytical framework to a diverse set of tens of thousands of natural product extracts and fractions which have been screened in the NCI‐60 panel by the NCI Program for Natural Product Discovery, with a long‐term goal to classify these products according to their potential antitumor mechanisms, and to provide a better understanding of how molecular characteristics of tumor cells play a role in their sensitivity to various agents.

## 5 Conclusions

We analyzed associations of the gene expression and mutation status of cancer‐related genes in the NCI‐60 cancer cell line panel with *in vitro* response to treatment with 1302 pure natural products and analogues that had been submitted to the NCI‐60 screening program. We identified several genes that were associated with tumor cell line response to specific categories of natural products. Associations of molecular features of cancer cell lines with response to natural products provided plausible hypotheses for the mechanisms of action of these compounds and identified clusters of different classes of natural compounds with potentially similar biological effects. This information will assist in future anticancer drug discovery from natural sources and in the design of new potential therapeutic agents.

## Conflict of interest

The authors declare no conflict of interest.

## Author contributions

JK, SN, LMY, YZ, LMM, JRE, and BROK conceived the study. JK, SN, AP, and YZ carried out the bioinformatic analysis of molecular genomic measures and their association with drug sensitivity, and drafted the manuscript. BROK participated in the design of the experimental study, oversaw the collection of natural product data and of *in vitro* molecular screen, and oversaw the completion the experimental work. TG participated in generation of experimental data including chemical annotations. JE provided bioinformatic support for generation of drug response measures and developed a classification of the natural products. JE, TG, and BROK participated in biological interpretation of the study results and in editing of the manuscript. HS contributed medical and biostatistical expertise throughout the study and assisted with interpretation of the study results. JF provided chemical interpretation of the study results. LMY and LMM oversaw the statistical and computational analysis of the data. All authors participated in the interpretation of study results and read, edited, and approved the final manuscript.

## Supporting information


**Fig. S1.** A flowchart demonstrating the selection of genes and SNVs for the analysis of association with response to 1302 natural products based on the OncoKB annotation.
**Fig. S2.** Hierarchical clustering of NCI‐60 cell lines based on the median log(GI50) values of 1302 natural products.
**Fig. S3.** Hierarchical clustering of 1302 natural products based on the median log(GI50) values of 59 NCI‐60 cell lines.
**Fig. S4.** Heatmap and two‐dimensional hierarchical clustering of NCI‐60 cell lines and 1302 natural products based on median log(GI50) values.
**Fig. S5.** Six biclusters (A–F) identified using the plaid model of biclustering of the log(GI50) matrix of NCI‐60 cell lines and 1302 natural products.
**Fig. S6.** Clustering of the chemical structures of the 77 natural products discussed in detail in the text.
**Fig. S7.** Comparison of chemical structures of the compound NSC 656161 and temozolomide.

**Table S1.** Chemical structures of the natural products discussed in the text.

**Table S2.** Detailed results for significant correlations of transcript expression with median log(GI50) values of natural products which satisfied FDR adjusted *P* < 0.05.

**Table S3.** Significant correlations of *SLFN11* transcript expression with median log(GI50) values of natural products, satisfying FDR adjusted *P* < 0.05.

**Table S4.** Natural compounds associated with *EPHX1* polymorphisms Y113H and H139R with *P < *0.05 prior to the FDR adjustment.

**Table S5.** Significant correlations of expression of the kinase genes *EGFR, KDR, FLT3, FLT4, KIT, SRC, PDGFRA, PDGFRB*, *NTRK1*, and *NTRK2* with median log(GI50) values of natural products satisfying FDR adjusted *P* < 0.05.

**Table S6.** Significant correlations of expression of the *ABC* family transporter genes with median log(GI50) values of natural products satisfying FDR adjusted *P* < 0.05.

**Table S7.** Significant associations of SNVs listed in OncoKB with response to the natural products.

**Table S8.** Additional protein‐changing SNVs in OncoKB genes which were significantly associated with differences in the NCI‐60 cell line response to the natural products.

## Data Availability

NCI‐60 expression and SNV data are publicly available from the CellMiner and CellMinerCDB online resources (https://discover.nci.nih.gov/cellminer/loadDownload.do; https://discover.nci.nih.gov/cellminercdb/) [33, 34]. Chemical structures of the natural products discussed in the text are provided in Table S1. Compounds described in this manuscript can be obtained, when supplies are present, from the DTP repository of pure compounds at the following address: https://dtp.cancer.gov/organization/dscb/obtaining/default.htm. For NCI‐60 data on pure natural products, such data can be requested by contacting the Natural Products Branch of the NCI (NCINatProdRep@mail.nih.gov).
