# Supplementary material for: Molecular genomic features associated with in vitro response of the NCI‐60 cancer cell line panel to natural products
Source: Mol Oncol. 2020 Nov 24;15(2):381–406. doi: 10.1002/1878-0261.12849 (PMC7858122; doi:10.1002/1878-0261.12849)
Supplement: Supplementary file 1 — Fig. S1. A flowchart demonstrating the selection of genes and SNVs for the analysis of association with response to 1302 natural products based on the OncoKB annotation. Fig. S2. Hierarchical clustering of NCI‐60 cell lines based on the median log(GI50) values of 1302 natural products. Fig. S3. Hierarchical clustering of 1302 natural products based on the median log(GI50) values of 59 NCI‐60 cell lines. Fig. S4. Heatmap and two‐dimensional hierarchical clustering of NCI‐60 cell lines and 1302 natural products based on median log(GI50) values. Fig. S5. Six biclusters (A–F) identified using the plaid model of biclustering of the log(GI50) matrix of NCI‐60 cell lines and 1302 natural products. Fig. S6. Clustering of the chemical structures of the 77 natural products discussed in detail in the text. Fig. S7. Comparison of chemical structures of the compound NSC 656161 and temozolomide. [file MOL2-15-381-s001.pdf]

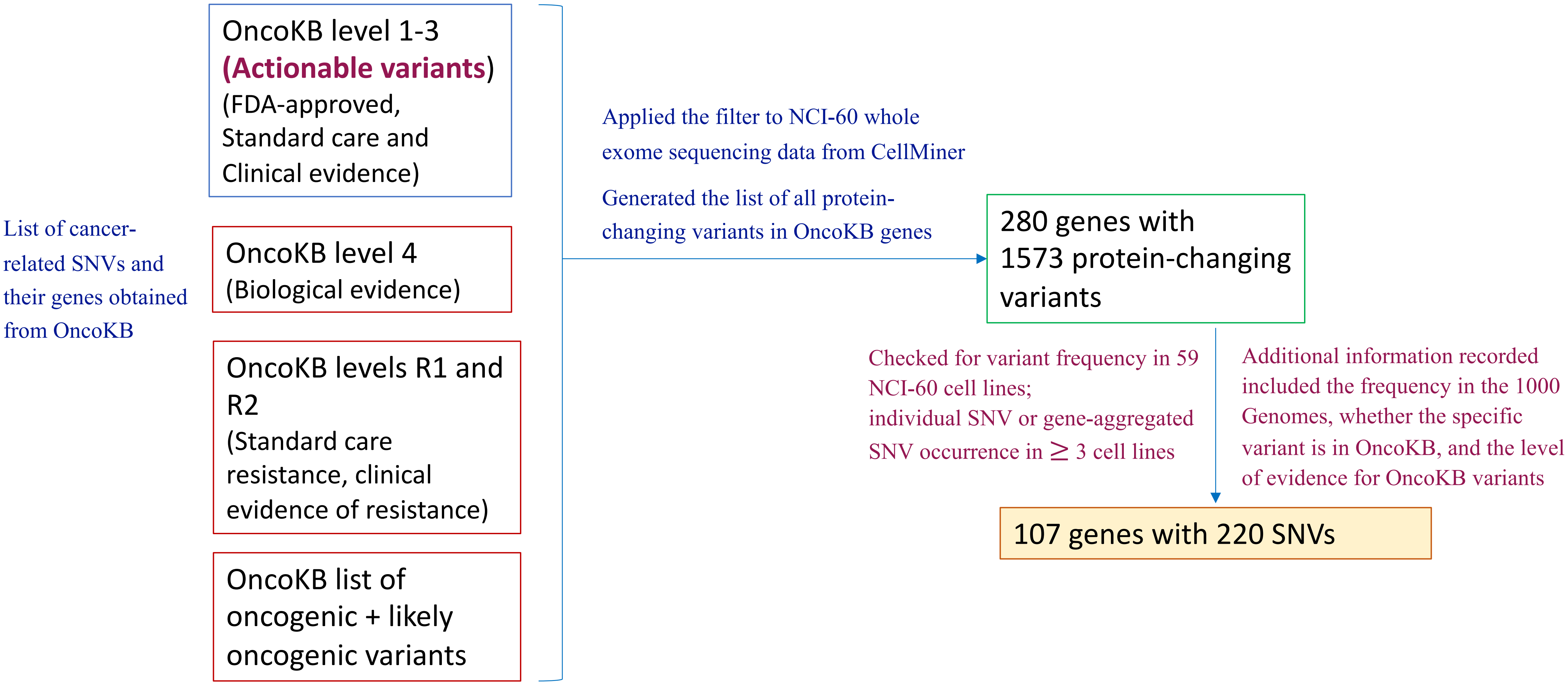

Figure S1. A flowchart demonstrating the selection of genes and single nucleotide variants for the analysis of association with response to 1,302 natural products based on the OncoKB annotation.

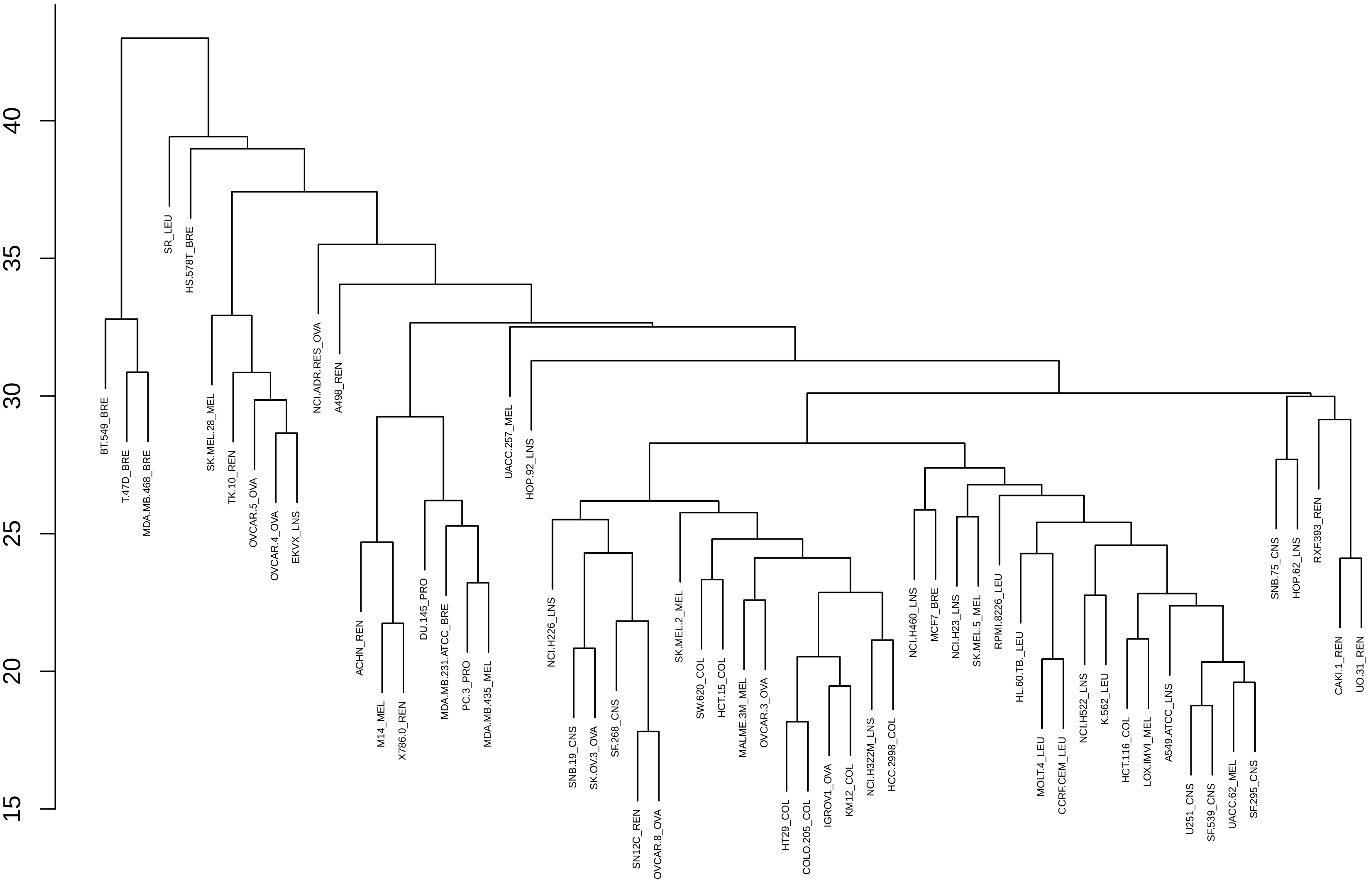

Figure S2. Hierarchical clustering of NCI-60 cell lines based on the median log(GI50) values of 1,302 natural products. The tree was inferred using the UPGMA ("average") method and was based on Euclidian distances. The tree is midpoint rooted and is presented as a hierarchical rectangular dendrogram. Clustering is identical to that presented in Fig. 2, providing an alternative display of identical clustering results. Tumor category information is appended to cell line names. **BRE,** breast; **CNS,** central nervous system; **COL,** colorectal; **LEU,** leukemia; **LNS,** lung; **MEL,** melanoma; **OVA,** ovarian; **PRO,** prostate; **REN,** renal.

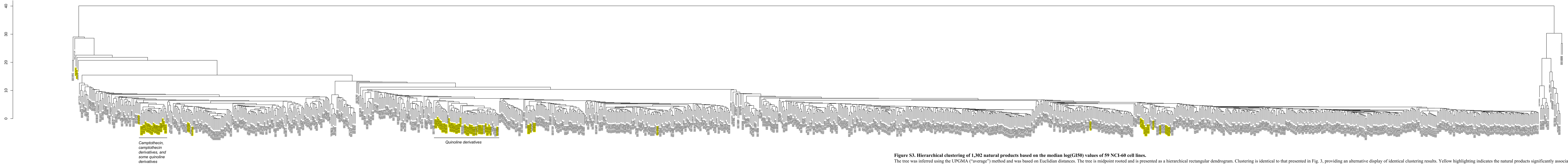

Figure S3. Hierarchical clustering of 1,302 natural products based on the median log(GI50) values of 59 NCI-60 cell lines.
The tree was inferred using the UPGMA ("average") method and was based on Euclidian distances. The tree is midpoint rooted and is presented as a hierarchical rectangular dendrogram. Clustering is identical to that presented in Fig. 3, providing an alternative display of identical clustering results. Yellow highlighting indicates the natural products significantly associated with expression of *SLFN11*.

Figure S4. Heatmap and two-dimensional hierarchical clustering of NCI-60 cell lines and 1,302 natural products based on median log(GI50) values.

Black color indicates cell line-natural compound pairs with missing GI50 values. Rows represent natural products. The vertical column on the right of the heatmap provides NSC numbers. Columns represent cell lines. Tumor category information is appended to cell line names. **BRE**, breast; **CNS**, central nervous system; **COL**, colorectal; **LEU**, leukemia; **LNS,** lung; **MEL**, melanoma; **OVA,** ovarian; **PRO**, prostate; **REN**, renal.

Color Key

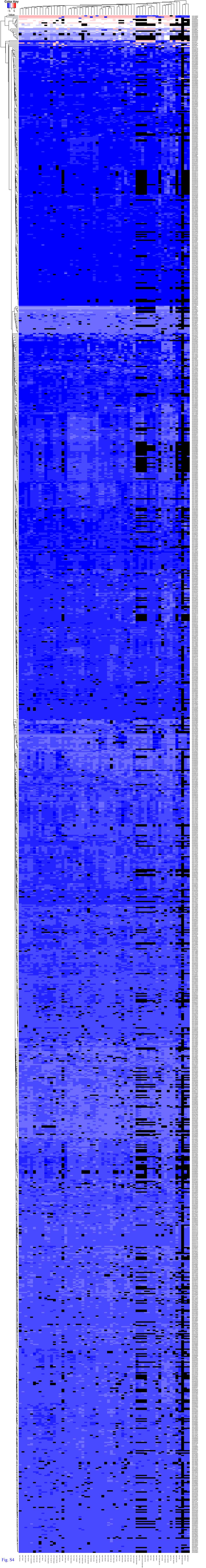

Fig. S4

Figure S5. Six biclusters (A-F) identified using the plaid model of biclustering of the log(GI50) matrix of NCI-60 cell lines and 1,302 natural products.

For the bicluster 1 (**A**) with 1,279 natural products and all 60 cell lines, only selected natural product names and cell lines are shown. Rows represent natural products. The vertical column on the left of each plot provides NSC numbers. Columns represent cell lines. Tumor category information is appended to cell line names. **BRE**, breast; **CNS**, central nervous system; **COL**, colorectal; **LEU**, leukemia; **LNS,** lung; **MEL**, melanoma; **OVA,** ovarian; **PRO**, prostate; **REN**, renal.

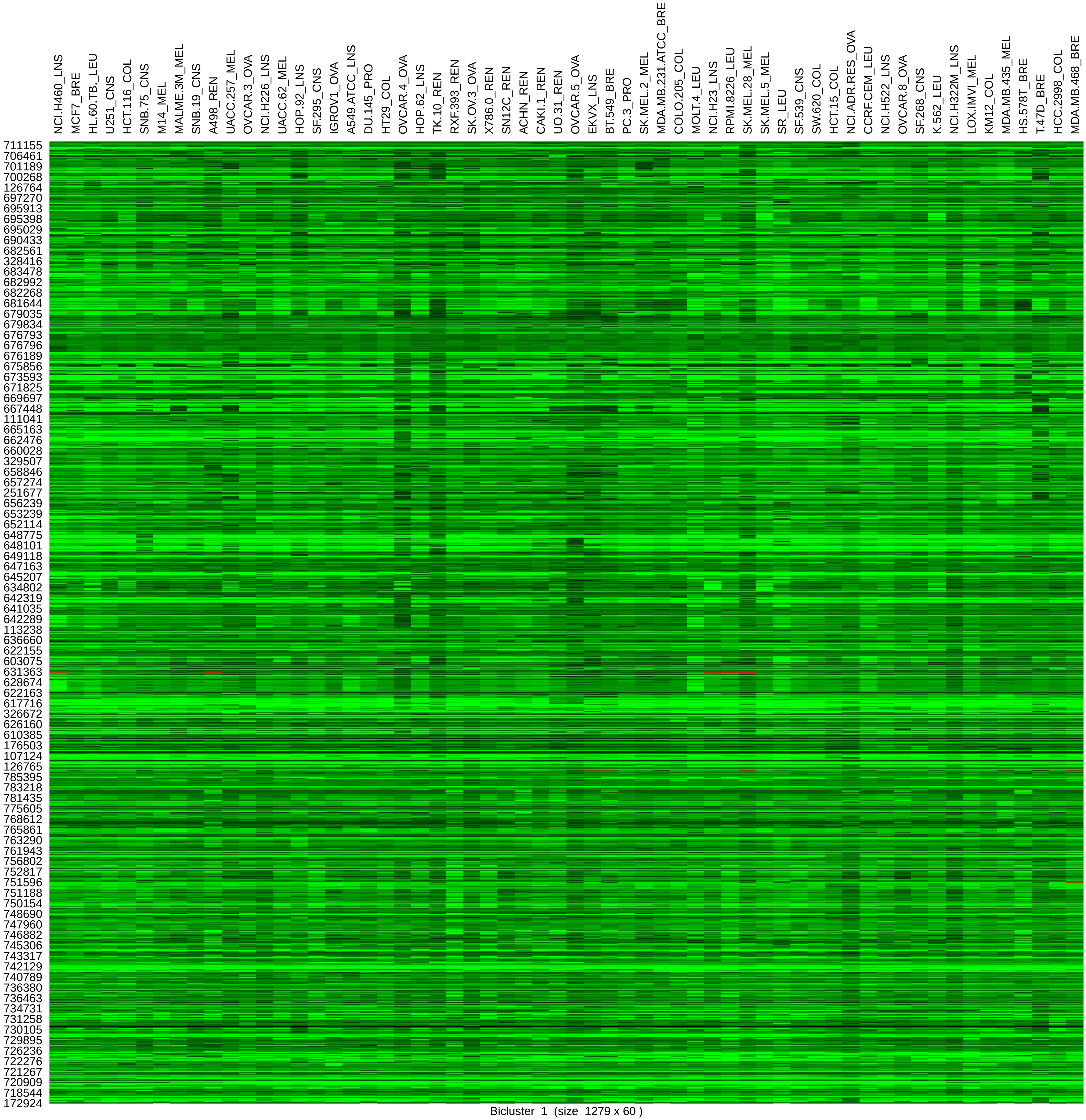
NCI.H460_LNS
MCF7_BRE
HL.60.TB._LEU
U251_CNS
HCT.116_COL
SNB.75_CNS
M14_MEL
MALME.3M_MEL
SNB.19_CNS
A498_REN
UACC.257_MEL
OVCAR.3_OVA
NCI.H226_LNS
UACC.62_MEL
HOP.92_LNS
SF.295_CNS
IGROV1_OVA
A549.ATCC_LNS
DU.145_PRO
HT29_COL
OVCAR.4_OVA
HOP.62_LNS
TK.10_REN
RXF.393_REN
SK.OV.3_OVA
X786.0_REN
SN12C_REN
ACHN_REN
CAKI.1_REN
UO.31_REN
OVCAR.5_OVA
EKVX_LNS
BT.549_BRE
PC.3_PRO
SK.MEL.2_MEL
MDA.MB.231.ATCC_BRE
COLO.205_COL
MOLT.4_LEU
NCI.H23_LNS
RPMI.8226_LEU
SK.MEL.28_MEL
SK.MEL.5_MEL
SR_LEU
SF.539_CNS
SW.620_COL
HCT.15_COL
NCI.ADR.RES_OVA
CCRF.CEM_LEU
NCI.H522_LNS
OVCAR.8_OVA
SF.268_CNS
K.562_LEU
NCI.H322M_LNS
LOX.IMVI_MEL
KM12_COL
MDA.MB.435_MEL
HS.578T_BRE
T.47D_BRE
HCC.2998_COL
MDA.MB.468_BRE
711155
706461
701189
700268
126764
697270
695913
695398
695029
690433
682561
328416
683478
682992
682268
681644
679035
679834
676793
676796
676189
675856
673593
671825
669697
667448
111041
665163
662476
660028
329507
658846
657274
251677
656239
653239
652114
648775
648101
649118
647163
645207
634802
642319
641035
642289
113238
636660
622155
603075
631363
628674
622163
617716
326672
626160
610385
176503
107124
126765
785395
783218
781435
775605
768612
765861
763290
761943
756802
752817
751596
751188
750154
748690
747960
746882
745306
743317
742129
740789
736380
736463
734731
731258
730105
729895
726236
722276
721267
720909
718544
172924
Bicluster 1 (size 1279 x 60)

Fig. S5A

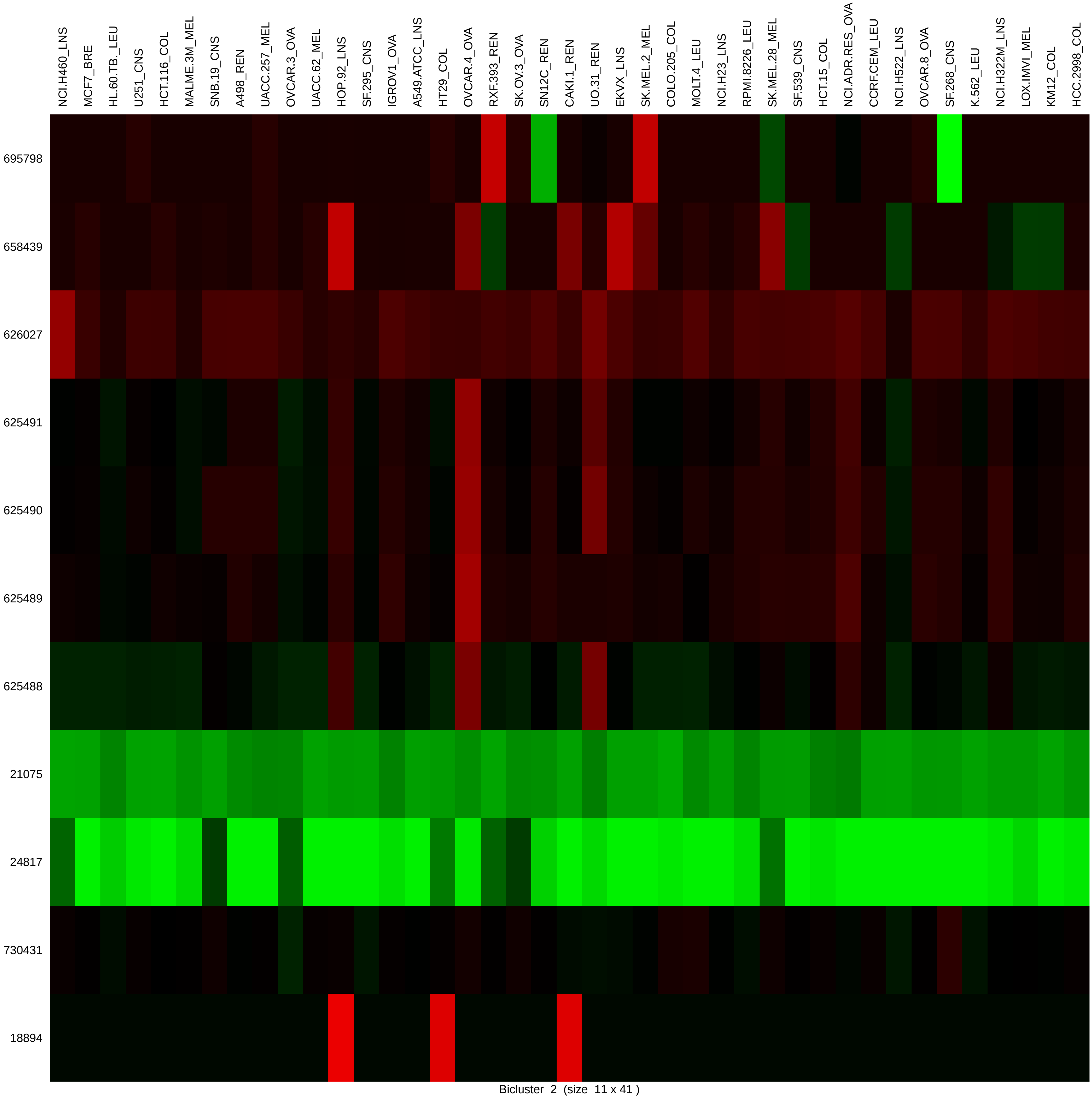
NCI.H460_LNS
MCF7_BRE
HL.60.TB._LEU
U251_CNS
HCT.116_COL
MALME.3M_MEL
SNB.19_CNS
A498_REN
UACC.257_MEL
OVCAR.3_OVA
UACC.62_MEL
HOP.92_LNS
SF.295_CNS
IGROV1_OVA
A549.ATCC_LNS
HT29_COL
OVCAR.4_OVA
RXF.393_REN
SK.OV.3_OVA
SN12C_REN
CAKI.1_REN
UO.31_REN
EKVX_LNS
SK.MEL.2_MEL
COLO.205_COL
MOLT.4_LEU
NCI.H23_LNS
RPMI.8226_LEU
SK.MEL.28_MEL
SF.539_CNS
HCT.15_COL
NCI.ADR.RES_OVA
CCRF.CEM_LEU
NCI.H522_LNS
OVCAR.8_OVA
SF.268_CNS
K.562_LEU
NCI.H322M_LNS
LOX.IMVI_MEL
KM12_COL
HCC.2998_COL
695798
658439
626027
625491
625490
625489
625488
21075
24817
730431
18894
Bicluster 2 (size 11 x 41)

Fig. S5B

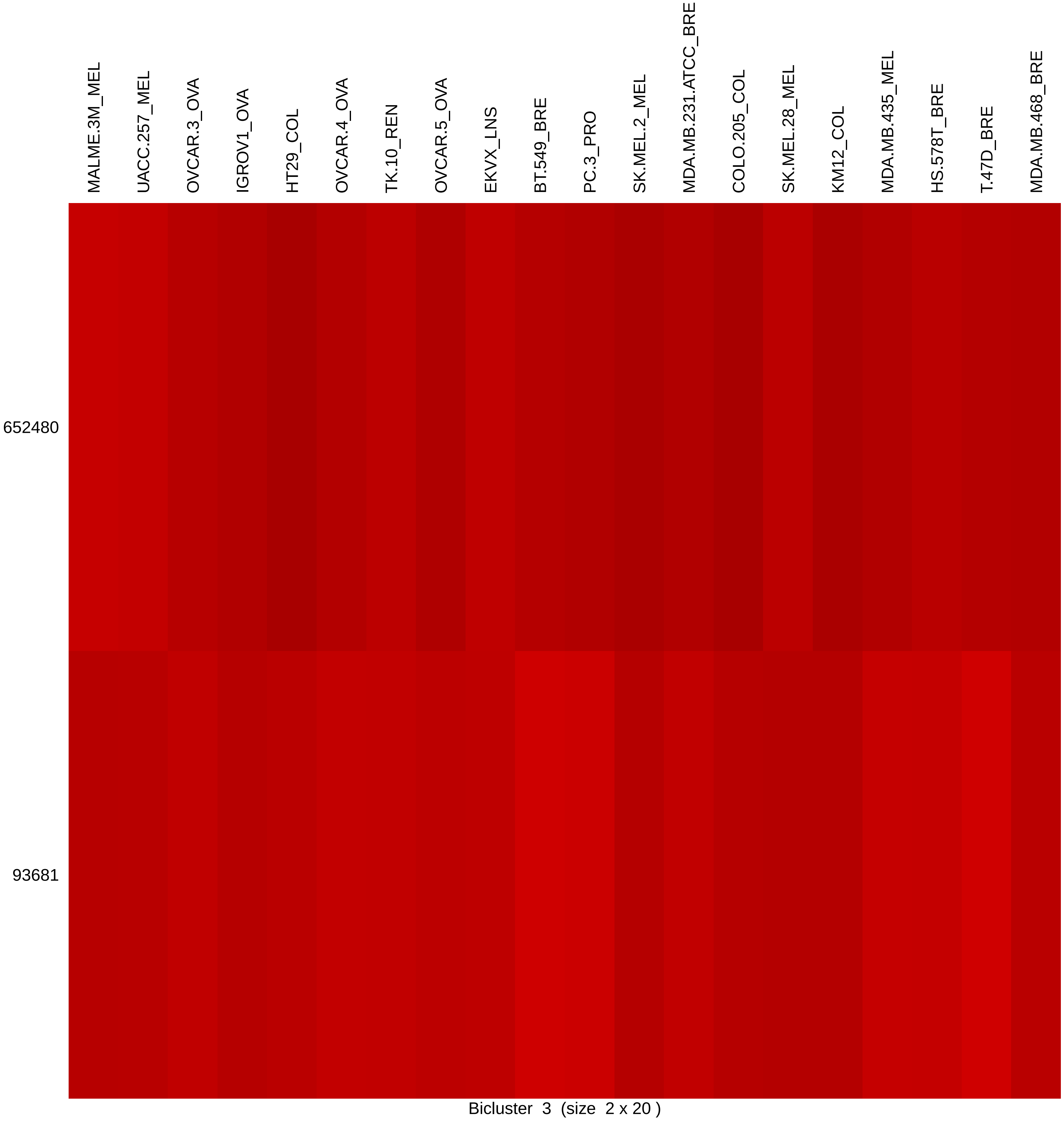
MALME.3M_MEL
UACC.257_MEL
OVCAR.3_OVA
IGROV1_OVA
HT29_COL
OVCAR.4_OVA
TK.10_REN
OVCAR.5_OVA
EKVX_LNS
BT.549_BRE
PC.3_PRO
SK.MEL.2_MEL
MDA.MB.231.ATCC_BRE
COLO.205_COL
SK.MEL.28_MEL
KM12_COL
MDA.MB.435_MEL
HS.578T_BRE
T.47D_BRE
MDA.MB.468_BRE
652480
93681
Bicluster 3 (size 2 x 20)

Fig. S5C

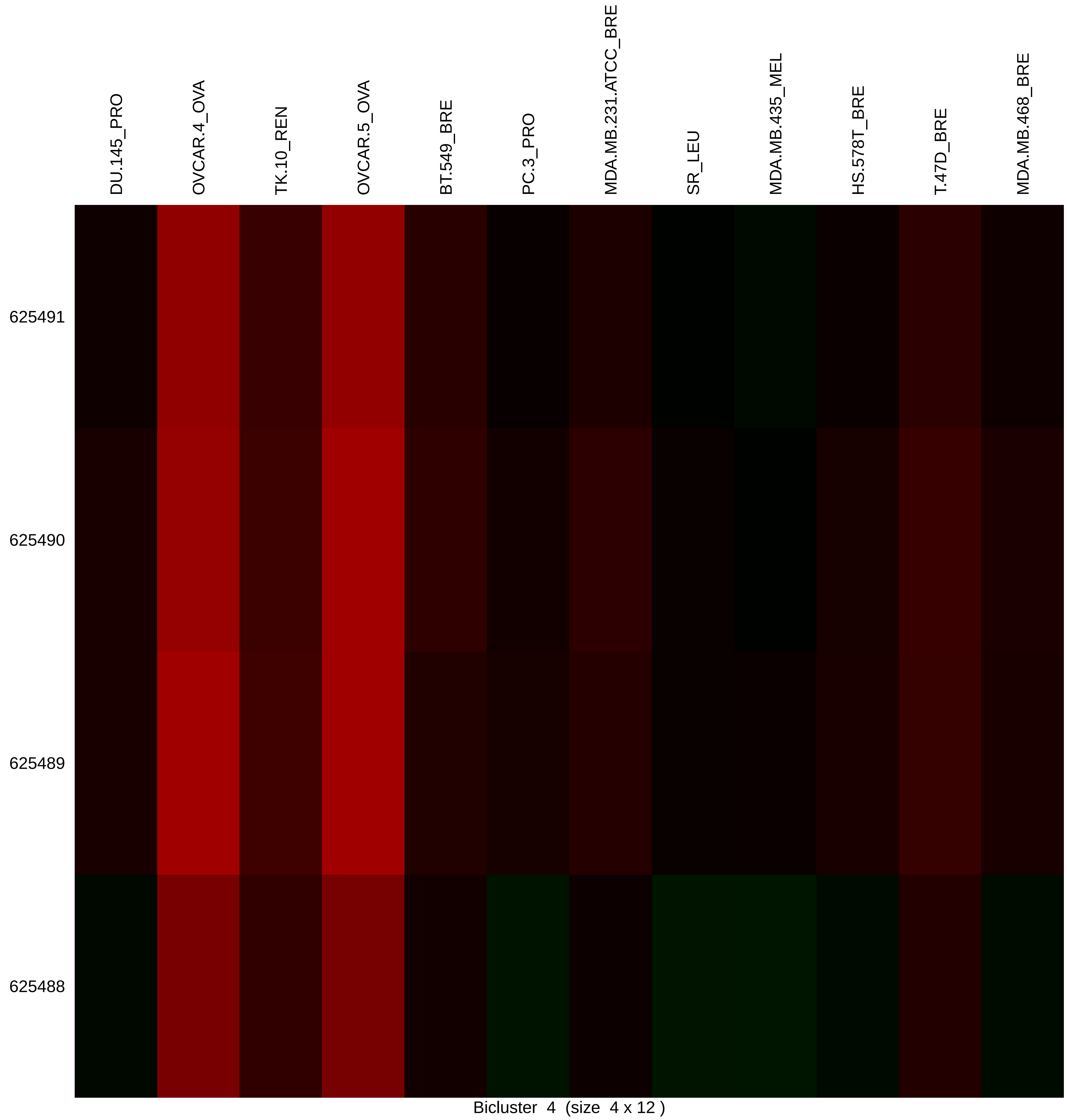
DU.145_PRO
OVCAR.4_OVA
TK.10_REN
OVCAR.5_OVA
BT.549_BRE
PC.3_PRO
MDA.MB.231.ATCC_BRE
SR_LEU
MDA.MB.435_MEL
HS.578T_BRE
T.47D_BRE
MDA.MB.468_BRE
625491
625490
625489
625488
Bicluster 4 (size 4 x 12)

Fig. S5D

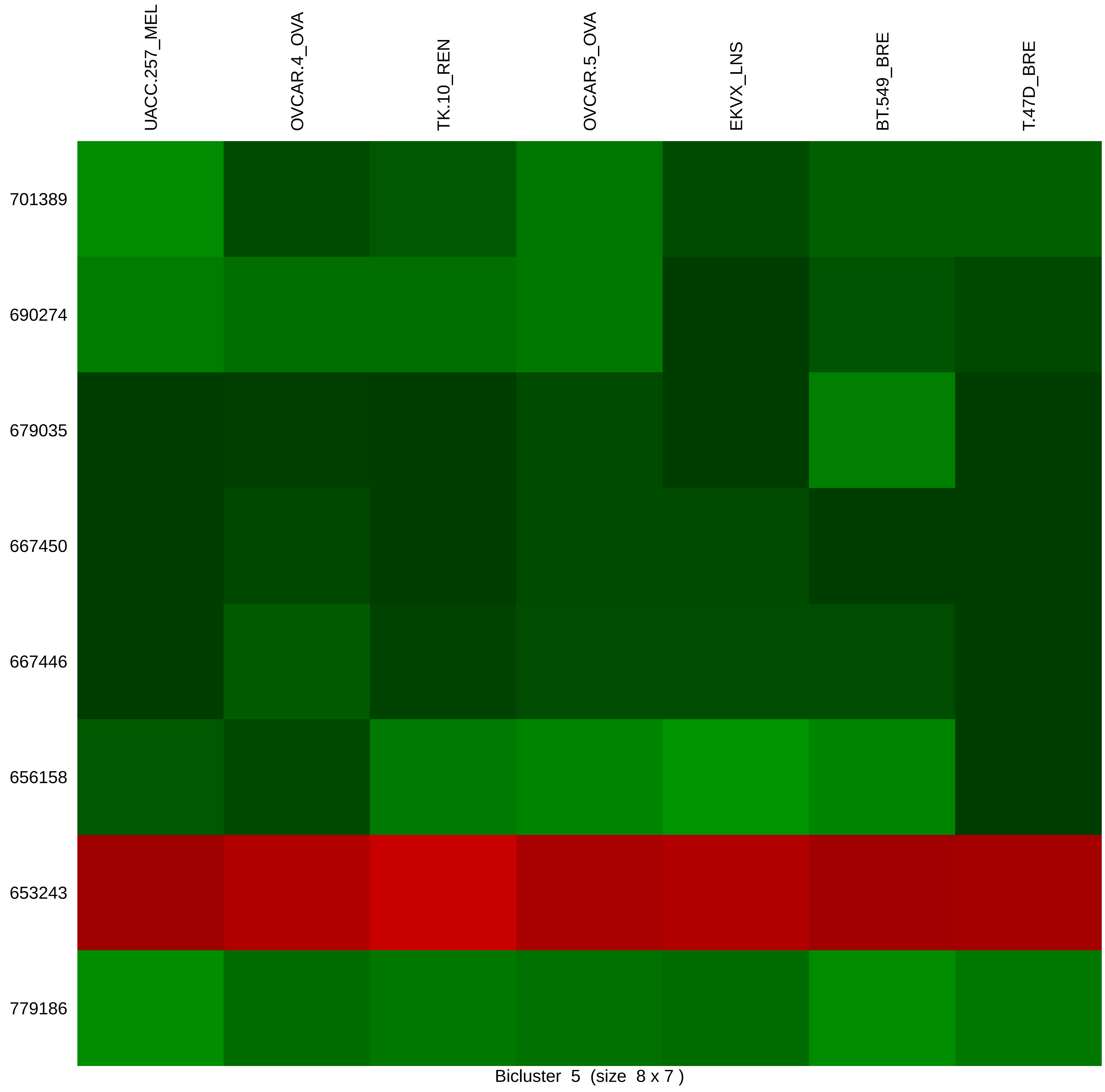
UACC.257_MEL
OVCAR.4_OVA
TK.10_REN
OVCAR.5_OVA
EKVX_LNS
BT.549_BRE
T.47D_BRE
701389
690274
679035
667450
667446
656158
653243
779186
Bicluster 5 (size 8 x 7)

Fig. S5E

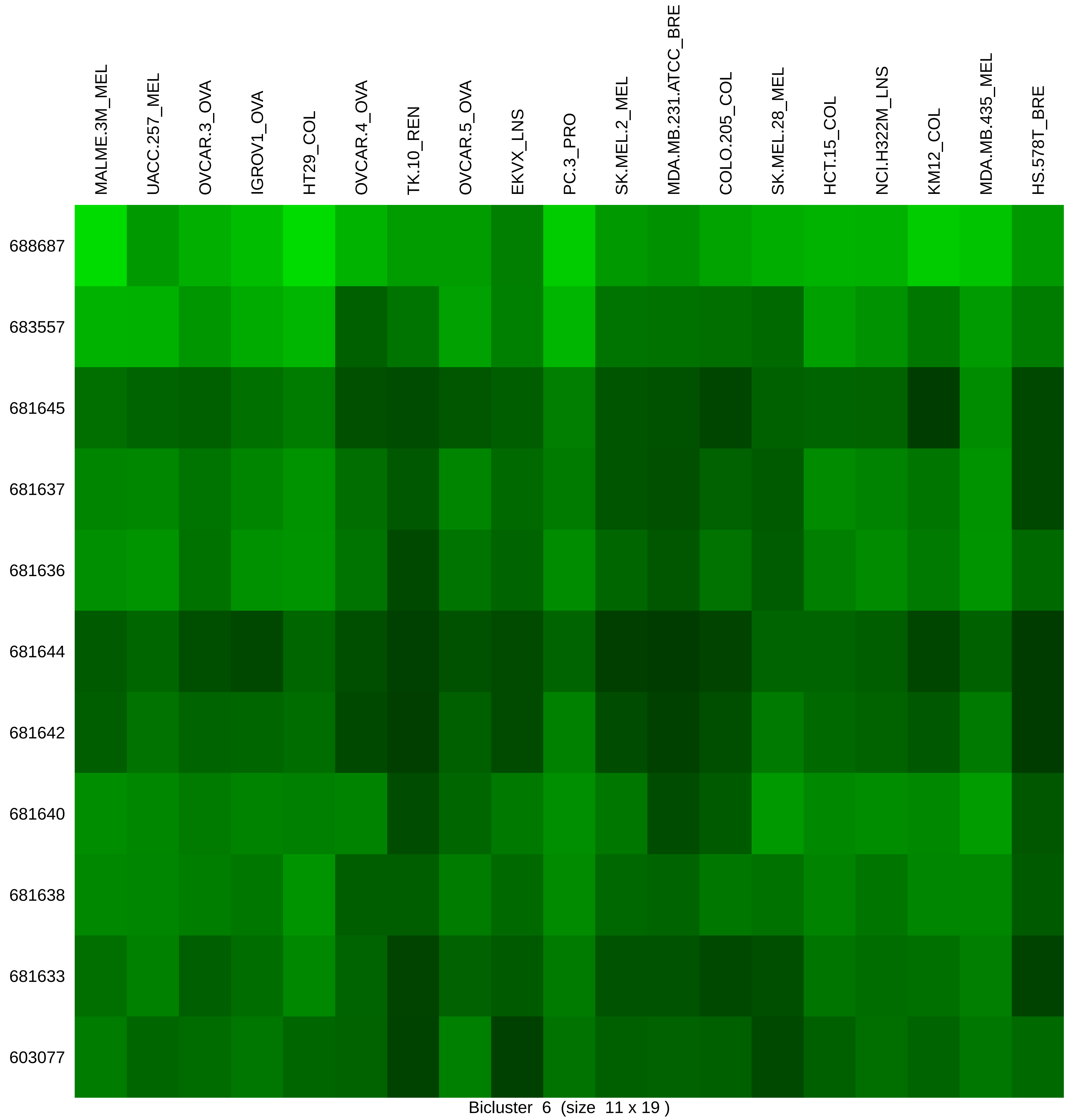
MALME.3M_MEL
UACC.257_MEL
OVCAR.3_OVA
IGROV1_OVA
HT29_COL
OVCAR.4_OVA
TK.10_REN
OVCAR.5_OVA
EKVX_LNS
PC.3_PRO
SK.MEL.2_MEL
MDA.MB.231.ATCC_BRE
COLO.205_COL
SK.MEL.28_MEL
HCT.15_COL
NCI.H322M_LNS
KM12_COL
MDA.MB.435_MEL
HS.578T_BRE
688687
683557
681645
681637
681636
681644
681642
681640
681638
681633
603077
Bicluster 6 (size 11 x 19)

Fig. S5F

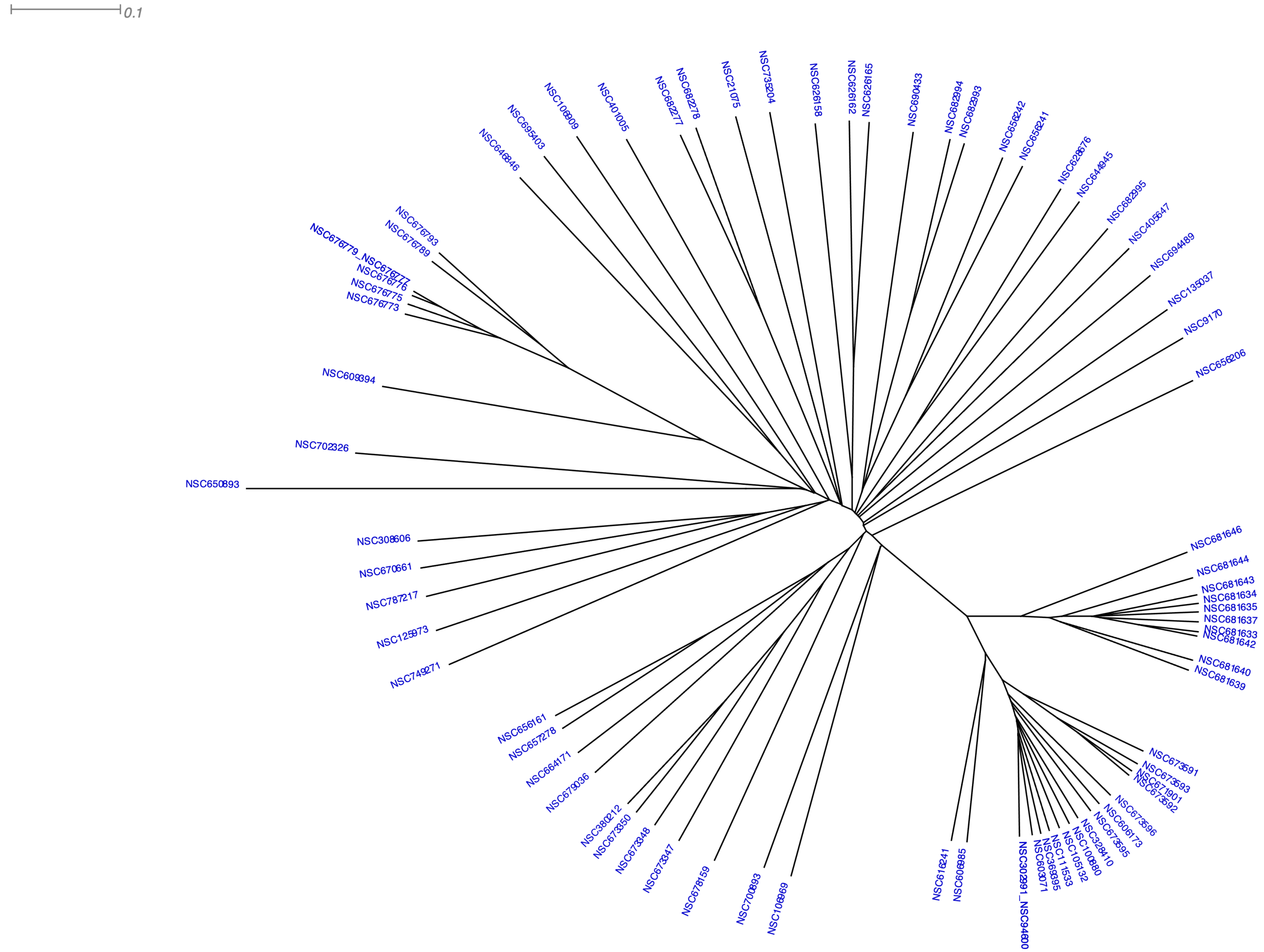

Figure S6. Clustering of the chemical structures of the 77 natural products discussed in detail in the text. The scale in the top left corner shows branch length units. Natural product structures are provided in Table S1.

Temozolomide

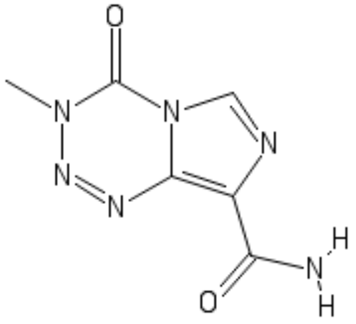

NSC656161

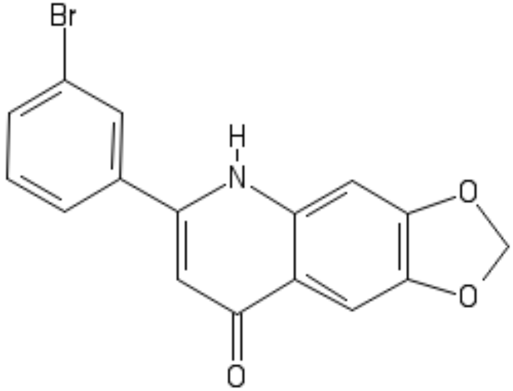

Figure S7. Comparison of the chemical structures of the compound NSC 656161 and temozolomide.

Chemical structures for both compounds were obtained from PubChem and visualized using PubChem Sketcher v.2.4.
